# A novel mechanism of antibody-mediated enhancement of flavivirus infection

**DOI:** 10.1371/journal.ppat.1006643

**Published:** 2017-09-15

**Authors:** Denise Haslwanter, Dieter Blaas, Franz X. Heinz, Karin Stiasny

**Affiliations:** 1 Center for Virology, Medical University of Vienna, Vienna, Austria; 2 Max F. Perutz Laboratories, Department for Medical Biochemistry, Medical University of Vienna, Vienna, Austria; NIH, UNITED STATES

## Abstract

Antibody-dependent enhancement of viral infection is a well-described phenomenon that is based on the cellular uptake of infectious virus-antibody complexes following their interaction with Fcγ receptors expressed on myeloid cells. Here we describe a novel mechanism of antibody-mediated enhancement of infection by a flavivirus (tick-borne encephalitis virus) in transformed and primary human cells, which is independent of the presence of Fcγ receptors. Using chemical cross-linking and immunoassays, we demonstrate that the monoclonal antibody (mab) A5, recognizing an epitope at the interface of the dimeric envelope protein E, causes dimer dissociation and leads to the exposure of the fusion loop (FL). Under normal conditions of infection, this process is triggered only after virus uptake by the acidic pH in endosomes, resulting in the initiation of membrane fusion through the interaction of the FL with the endosomal membrane. Analysis of virus binding and cellular infection, together with inhibition by the FL-specific mab 4G2, indicated that the FL, exposed after mab A5- induced dimer-dissociation, mediated attachment of the virus to the plasma membrane also at neutral pH, thereby increasing viral infectivity. Since antibody-induced enhancement of binding was not only observed with cells but also with liposomes, it is likely that increased infection was due to FL-lipid interactions and not to interactions with cellular plasma membrane proteins. The novel mechanism of antibody-induced infection enhancement adds a new facet to the complexity of antibody interactions with flaviviruses and may have implications for yet unresolved effects of polyclonal antibody responses on biological properties of these viruses.

## Author summary

Antibodies are an important component of antiviral host responses and their binding to the surface of virus particles usually leads to neutralization of viral infectivity. In some instances, however, antibodies at sub-neutralizing concentrations can enhance infection of certain cells, because they facilitate the uptake of infectious virus-antibody complexes through interactions with antibody-specific cellular receptors (Fcγ receptors). This mechanism is designated antibody-dependent enhancement of infection and implicated in the pathogenesis of dengue and possibly Zika virus infections, both mosquito-transmitted flaviviruses. Here we describe a novel mechanism of infection enhancement by antibodies that is independent of interactions with Fcγ receptors, using another important human-pathogenic flavivirus, tick-borne encephalitis virus. We demonstrate that binding of a specific antibody to the envelope protein E at the viral surface promotes the exposure of a structural element that interacts with the lipids of the cellular plasma membrane, thus increasing infection. Our study provides new insights into mechanisms that potentially modulate the antiviral effects of antibody populations present in post-infection sera.

## Introduction

Flaviviruses are small, enveloped viruses that cause significant human disease worldwide, including the mosquito-borne dengue, Zika, West Nile, Japanese encephalitis, and yellow fever viruses as well as tick-borne encephalitis virus (TBEV) [1]. Despite intensive research, the hunt for specific flavivirus receptors has yielded diverse results. A plethora of molecules at the plasma membranes of different cells have been shown to interact with flaviviruses and were proposed to function as attachment factors, but *bona fide* entry receptors are still ill-defined (reviewed in [2, 3]). The reported data are quite varied, suggesting that molecules involved in flavivirus cell attachment and entry differ among viruses and cells [4]. In most instances, the major envelope protein E (which mediates viral membrane fusion upon receptor-mediated endocytosis) has been implicated in such interactions. More recently, cellular lipid receptors, (TIM (T cell immunoglobulin mucin domain) and TAM (Tyro3, Axl and Mer) receptor families) that recognize lipids in the viral membrane, have been shown to mediate flavivirus attachment and entry in certain instances [5, 6].

**Fig 1:**
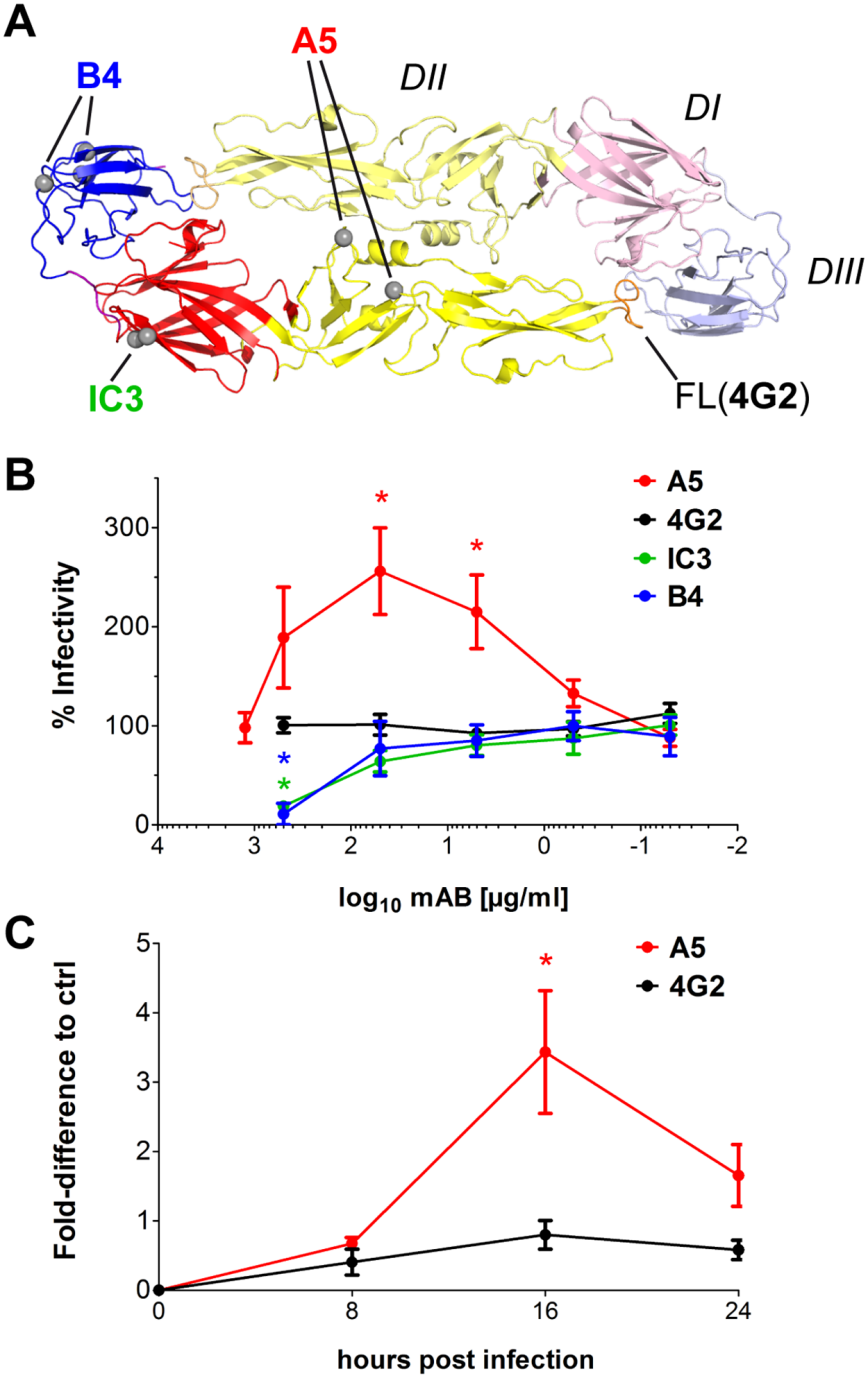
Mab A5-induced enhancement of TBEV infectivity and growth. (A) Ribbon diagram of the TBEV E dimer (PDB: 1SVB, [11]). Grey spheres indicate sites of amino acid mutations that affected binding of mabs [58, 59, 81, 93]. (B) Focus reduction neutralization tests (FRNTs) with TBEV on HeLa cells using four different E-specific mabs (red—A5, black—4G2, green—IC3, blue—B4). The y-axis indicates percent infectivity obtained in the absence of mabs. Data represent the mean +/- standard error of the mean (SEM) of at least four independent experiments. (C) Enhancement of TBE virus infectivity in HeLa cells at different time points post infection. Cells were infected with TBEV and TBEV-mab mixtures (A5- red, 4G2—black), and at the indicated times post infection, aliquots were used for focus formation assays. The y-axis indicates the fold-difference of focus forming units (FFU) relative to the control without mab. Data represent the mean +/- SEM of four independent experiments. Infectivity of TBEV in the absence and presence of different mabs was compared with ANOVA followed by Dunnett’s multiple comparisons test. *, p < 0.05.

Flaviviruses are assembled at the endoplasmic reticulum as immature virions [7], in which the E proteins are associated with the prM protein (precursor of M) as heterodimers that form trimeric spikes [8]. During exocytosis, prM is processed by the cellular enzyme furin, giving rise to mature virions that contain the small M protein and E homodimers [9] covering the viral surface in a herringbone-like arrangement [10]. Each E monomer has three distinct domains (domain I, II, III; Fig 1A), connected by short flexible linkers [11]. Domain II provides most of the intra-dimeric contacts and contains the conserved fusion loop (FL) at its distal end. In the dimer, the FL is buried in a pocket built by domains I and III of the neighboring subunit (Fig 1A). Most flavivirus E proteins have a single N-linked glycan attached to domain I, while the dengue E proteins have an additional glycan in domain II [12].

In addition to direct virus-cell interactions, antibodies bound to virus particles can mediate attachment of such immune complexes to Fcγ receptor (FcγR)-bearing cells like monocytes, macrophages and dendritic cells, leading to endocytosis and—in the case of incomplete virus neutralization—to enhancement of infection [13–15]. This mechanism of antibody-dependent enhancement (ADE) is a well-documented phenomenon of flavivirus infections (reviewed in [16, 17]), but was also observed for many other viruses that are able to replicate in FcγR-positive cells [18]. ADE has been implicated in the pathogenesis of severe forms of dengue virus infections and is speculated to contribute to congenital Zika virus infections [17, 19, 20], but the precise role of ADE in human infections is still debated (reviewed in reference [21]).

Recent evidence indicates that the proteins in the flavivirus envelope are in dynamic motion (referred to as “virus breathing”), best described as an ensemble of conformations around equilibrium (reviewed in [22]). These structural dynamics of the virus surface transiently expose sites in E that would be inaccessible in a static virus particle with a closed shell of 90 E dimers in a herringbone-like arrangement, as determined by cryo EM [10]. Structural analyses, however, have also revealed temperature-dependent changes of the viral envelope for certain dengue virus strains [23, 24], which can result in the exposure of cryptic epitopes and their recognition by certain mabs, leading to virus neutralization [25, 26]. Through binding to such transiently accessible sites, antibodies, and possibly other ligands [27], can lock E in different conformational states within the virion [25].

The structural dynamics of flaviviruses reflects the conformational flexibility of E, which is required during particle maturation as well as during viral membrane fusion. It is primarily made possible by flexible linker elements between each of the domains (Fig 1A). For membrane fusion, the acidic pH of endosomes triggers the dissociation of E dimers and thus exposes the FL, allowing for its interaction with the endosomal membrane and the initiation of fusion. Membrane merging is then driven by an irreversible structural change of E that converts it into a stable post-fusion trimer (reviewed in [28, 29]).

In this work, we describe a novel mechanism underlying antibody-mediated infection enhancement of TBEV in FcγR-negative cells that is dependent on FL-lipid interactions. We show that a mab recognizing an epitope at the E dimer interface, leads to the dissociation of the E dimer and thus to exposure of the FL. The FL then mediates direct attachment to the plasma membrane (and liposomal membranes) and thus increases the infectivity of TBE virus in FcγR-negative cells. Such a mechanism of antibody-mediated enhancement of infection has not yet been described for flaviviruses and adds a new facet to the potential effects of antibodies in flavivirus infections.

## Results

### Antibody-mediated enhancement of infection

In the course of focus-reduction neutralization tests in HeLa-H1 (HeLa) cells, we observed a paradoxical infection-enhancing effect with one mab (A5) out of a set of mabs specific for different antigenic sites in the E protein of TBEV (Fig 1A). As seen in Fig 1B, mab A5 did not neutralize the virus even at the highest concentration of 1,250 μg/ml, but rather caused a significant increase (up to 2.5-fold) of infectivity. This property was not seen with mabs directed to other sites in E that either neutralized the virus (IC3, B4) or had no effect on infectivity (4G2) (Fig 1B). Measuring the increase of virus production as a function of time post infection in HeLa cells revealed that mab A5-mediated infection enhancement was most prominent at early times of virus replication. After pre-incubation of the virus (MOI 0.25) with mab A5, the maximum increase of virus yield (approximately 3-fold) was reached at 16 hours post infection (Fig 1C), which was also the time point of peak virus production in the absence of antibody. No significant enhancement effect was observed with the control mab 4G2 (Fig 1C). Since HeLa cells do not express FcγRs [30], these data suggest that the binding of mab A5 increased the infectivity of TBEV by an FcγR-independent mechanism.

As suggested by blocking ELISAs with human sera (Materials and methods), mab A5-like antibodies can also be induced in the course of natural TBEV infections, albeit in relatively rare instances. Only one (serum #4) out of 30 sera was found to be positive in this assay (54% mab A5 blocking activity, S1 Table). Since several samples had higher overall TBEV-specific IgG titers than serum #4, but were negative in mab A5 blocking (S1 Table), this property does not appear to be linked to the total amount of specific antibodies present but to reflect the heterogeneity of antibody compositions in polyclonal sera from different individuals.

### Antibody-mediated binding to cells and liposomes

**Fig 2:**
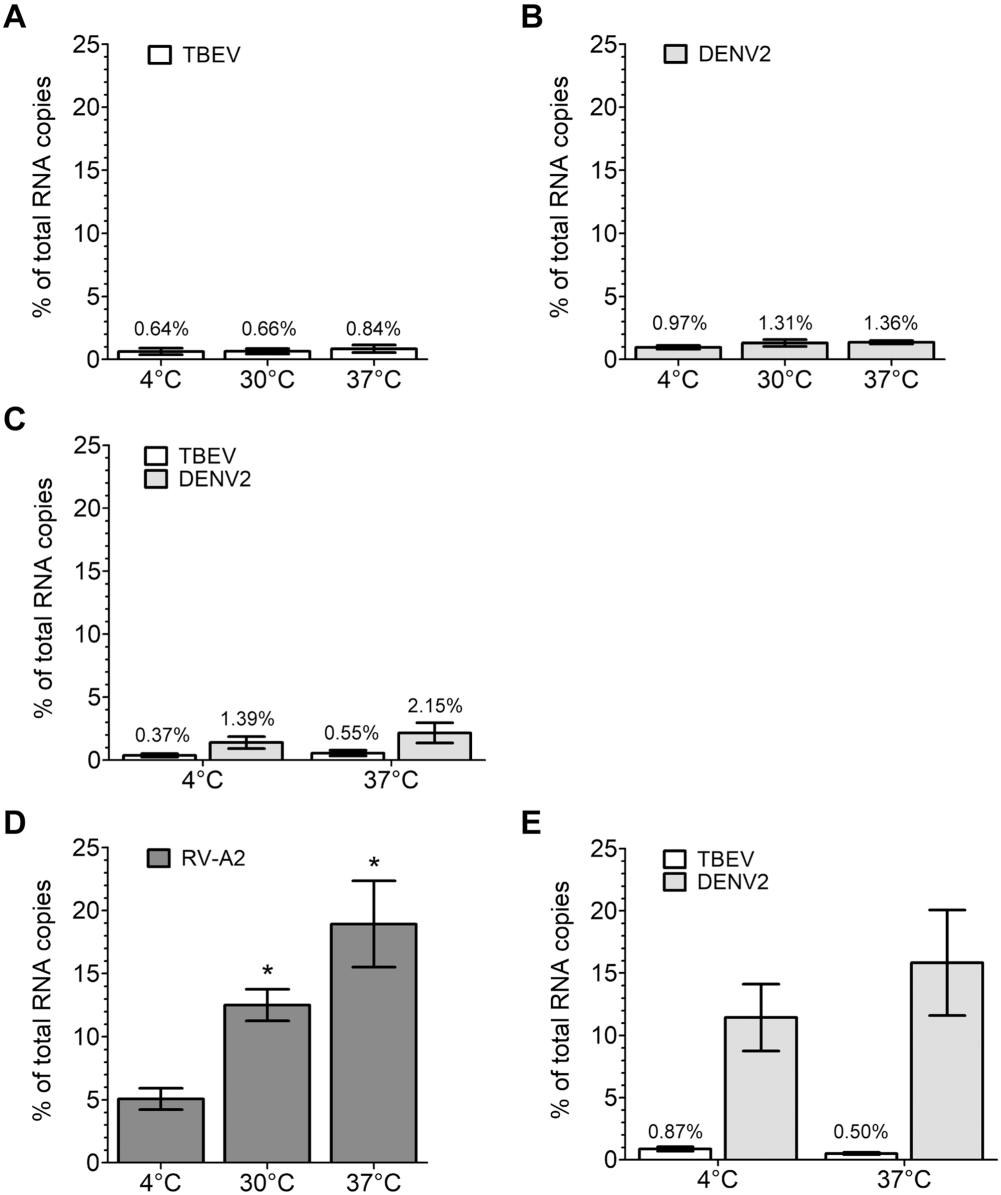
Virus-cell binding assays. (A-B) Binding of virus to HeLa cells. (A) TBEV, (B) DENV. (C) Binding of TBEV (white columns) or DENV (grey columns) to human foreskin fibroblasts (HFFs). (D) Binding of RV-A2 to HeLa cells. (E) Binding of TBEV (white columns) or DENV (grey columns) to immature monocyte-derived dendritic cells (moDCs). Viruses were allowed to attach to cells for 1 hour at the temperatures as indicated on the x-axis. The y-axis indicates percent bound virus relative to input virus using RNA copy numbers determined by quantitative PCR (qPCR). Data represent the mean +/- SEM of at least three independent experiments. For each virus, the amounts of bound virus at 30°C and/or 37°C were compared to those obtained at 4°C with ANOVA followed by Dunnett’s multiple comparisons test (A,B,E) or Student’s t-test (C,D). *, p < 0.05.

A possible explanation for the observed enhancement of infectivity is the facilitation of viral cell attachment by mab A5. Therefore, we first quantified virus attachment to cells in the absence of antibodies by using qPCR (Materials and methods). Given that HeLa cells were efficiently infected by TBEV, binding of the virus to HeLa cells was surprisingly low (less than 1% of input virus), irrespective of the temperature used (4, 30, and 37°C) (Fig 2A). To analyze whether this was a specific property of TBEV, we performed the same experiment with dengue virus (serotype 2, DENV2, strain New Guinea C), which also replicates efficiently in HeLa cells. Although binding was slightly higher than for TBEV, the values were again remarkably low (less than 1.5% of input virus) (Fig 2B). A similar picture was obtained in binding experiments with primary human foreskin fibroblasts (HFFs), indicating that the low degree of virus attachment was not restricted to transformed cell lines such as HeLa cells (Fig 2C).

To validate these results and our experimental system, we used two approaches. First, we analyzed binding/internalization of human rhinovirus type 2 (RV-A2) to HeLa cells, a system with well-defined and specific receptors (i.e. members of the low-density lipoprotein receptor superfamily; [31]). As seen in Fig 2D, binding/internalization of RV-A2 was dramatically more efficient than that of the flaviviruses tested. It strongly increased with temperature, as described in the literature [32, 33]. Secondly, we quantified binding of TBE and DEN2 viruses to immature monocyte-derived dendritic cells (moDC), expressing DC-SIGN (S1 Fig), which has been shown to be a specific attachment factor for dengue viruses [34]. Consistent with the potential of dengue viruses to interact with DC-SIGN, efficient binding of DENV2 to immature moDCs was observed, whereas binding of TBEV was as low as on HeLa cells (Fig 2E).

**Fig 3:**
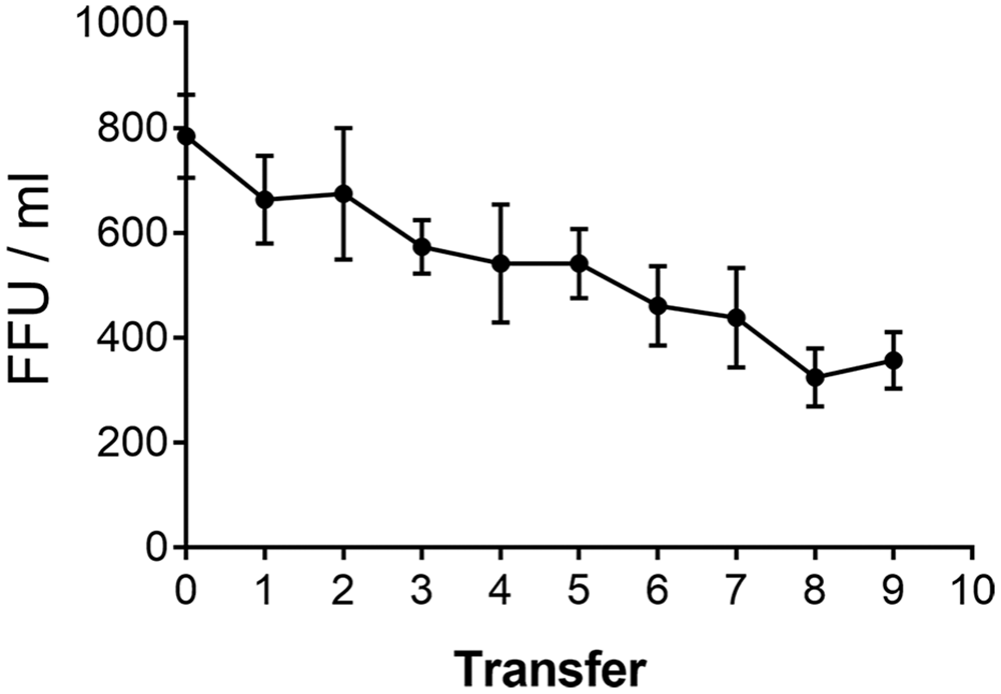
Serial transfer of infectivity using TBEV and HeLa cells. TBEV was allowed to bind to HeLa cells for 30 minutes at 4°C (0). Unbound virus was then transferred onto fresh pre-chilled cells for a new round of binding. The procedure was repeated nine times and the number of foci was determined after shifting the cells to 37° as described in Materials and Methods. Data represent the mean +/- SEM of at least four independent experiments.

To assess possible implications of the low levels of cellular attachment (Fig 1) on measurements of infectivity, we performed serial transfer experiments in a standard focus assay format (Materials and methods). Consistent with the low degree of binding (see Fig 2), the number of foci declined only moderately from step to step (Fig 3), indicating that the usual determination of infectious units in virus-containing samples will yield a gross underestimate of the real number of infectious virus particles present.

**Fig 4:**
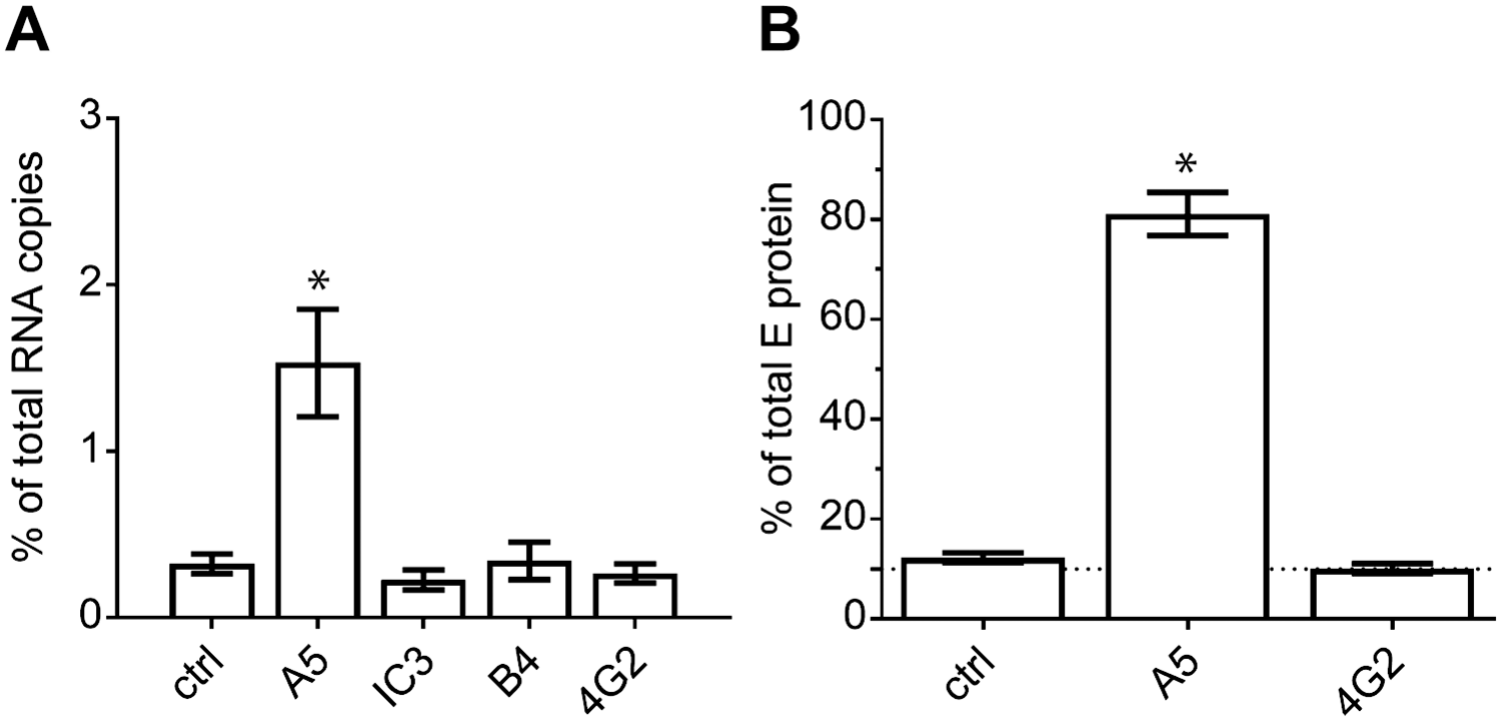
Mab A5-enhanced TBEV binding to cells and liposomes. (A) TBEV was allowed to attach to HeLa cells for 1 hour at 4°C in the absence or presence of mabs (as indicated on the x-axis). The y-axis indicates percent bound virus relative to input virus using RNA copy numbers determined by qPCR. (B) TBEV was allowed to attach to liposomes for 20 minutes at 37°C in the absence or presence of mabs (as indicated on the x-axis). The y-axis indicates percent bound virus relative to input virus using the amounts of E protein determined by quantitative ELISA. Data represent the mean +/- SEM of at least three independent experiments. The amounts of bound TBEV-mab complexes were compared to those obtained with the control (ctrl, TBEV without mabs) with ANOVA followed by Dunnett’s multiple comparisons test. *, p < 0.05.

We next investigated the influence of mab A5 on TBEV attachment to HeLa cells. In contrast to other antibodies, mab A5 increased cell binding significantly (approximately 4-to-5-fold, Fig 4A), supporting the hypothesis, that enhancement of infectivity was caused by facilitation of cell attachment. Mechanistically, one might imagine that interaction of the antibody with the virus surface leads to structural rearrangements [25] and the exposure of sites in E that allow interactions with yet unknown cellular protein receptors. If this were the case, no such effects should be seen in interactions with liposomes that consist of a mere lipid membrane. Results of binding experiments with liposomes are shown in Fig 4B. They clearly demonstrate that mab A5 strongly enhances binding to lipid membranes, suggesting that the phenomenon of A5-mediated enhancement of infectivity is due to direct interaction of E with the lipids of the plasma membrane.

### Mab-A5-mediated enhanced cell binding is independent of FcγRs

**Fig 5:**
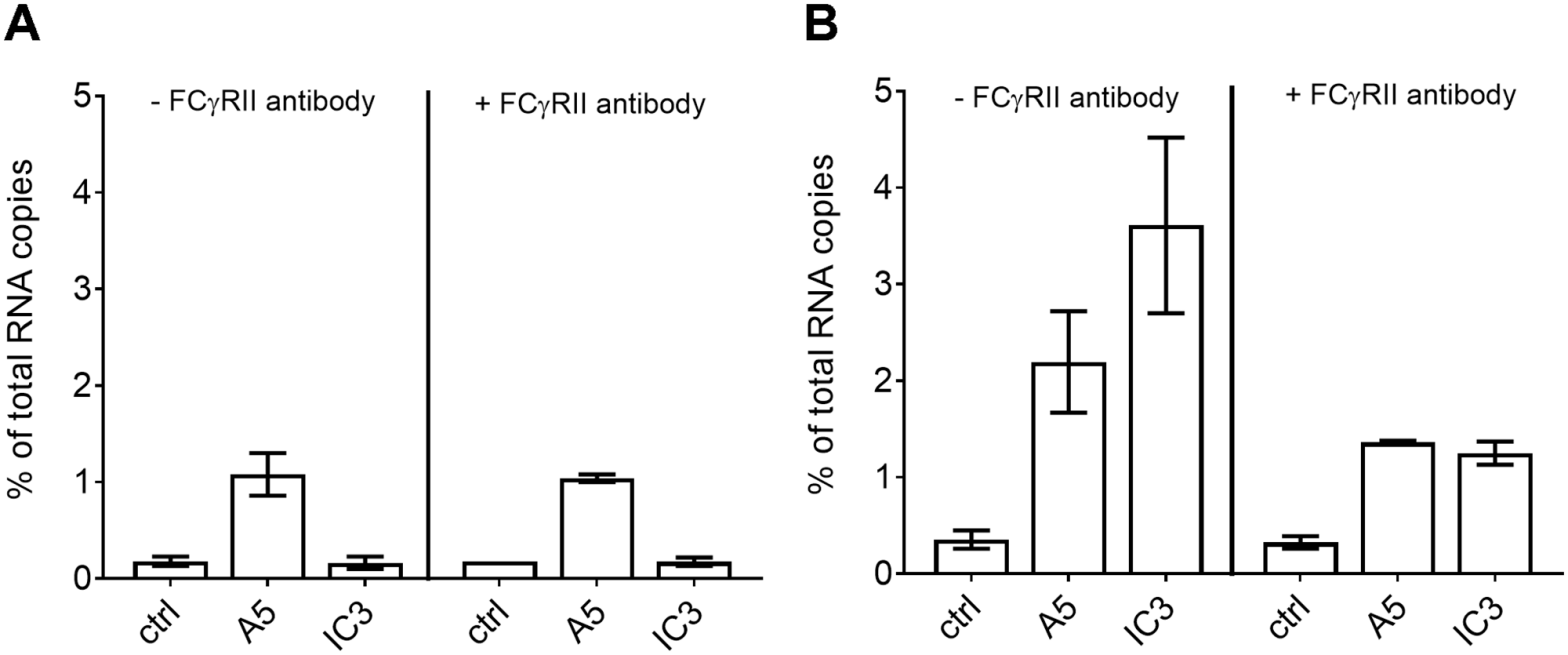
Binding of TBEV to HeLa cells without and after pre-treatment with an FcR-specific antibody. (A) Binding of TBEV in the absence or presence of mabs A5 and IC3 to HeLa cells without pretreatment (left panel) and after pretreatment with the anti-Fcγ RII/CD32a mab (right panel). (B) Binding of TBEV in the absence or presence of mabs A5 and IC3 to K562 cells without pretreatment (left panel) and after pretreatment with the anti-Fcγ RII/CD32a mab (right panel). The y-axis indicates percent bound virus relative to input virus using RNA copy numbers determined by qPCR. Data represent the mean and range of two independent experiments.

To verify that binding of TBEV to HeLa cells in the presence of mab A5 did not involve mab-FcγR-interactions, we performed blocking experiments with the FcγR-specific mab CD32 [35], using the FcγR-positive K562 cell line as a control [36]. Consistent with the lack of FcγRs in HeLa cells [30], the pre-incubation with mab CD32 had no effect on mab A5-mediated virus attachment to these cells (Fig 5A). In contrast, and as expected, mab A5-mediated binding of TBEV to K562 cells was reduced upon pre-treatment of the cells with mab CD32 (Fig 5B).

### Mab A5 induces dissociation of E and FL exposure

**Fig 6:**
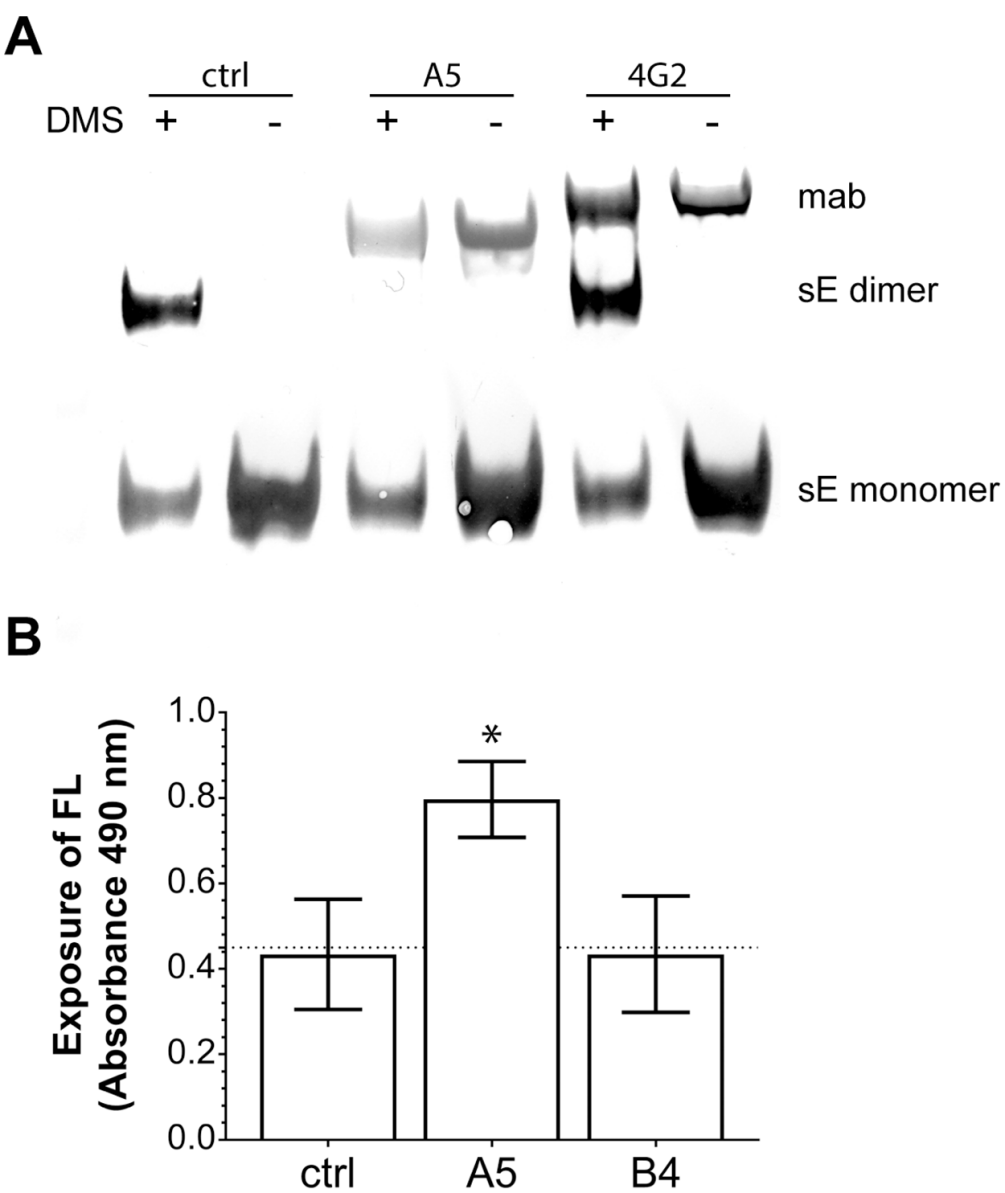
Mab A5-induced dissociation of E. (A) Chemical cross-linking with dimethyl suberimidate (DMS), SDS-PAGE and Western blotting of a soluble form of TBEV E (sE) with and without mabs. The protein bands corresponding to sE monomers, sE dimers and mabs are labeled. Lane 1: sE plus DMS, lane 2: sE without DMS, lane 3: sE plus DMS in the presence of mab A5, lane 4: sE without DMS in the presence of mab A5, lane 5: sE plus DMS in the presence of mab 4G2, lane 6: sE without DMS in the presence of mab 4G2. A representative experiment out of three is shown. (B) Exposure of the FL detected by 4-Layer ELISA with TBEV and mabs in the absence of detergent. TBEV was captured with a polyclonal anti-TBEV IgG. After the addition of buffer (control, ctrl) or mabs (A5, B4), the biotin-labeled mab 4G2 was used to measure the exposure of the FL. Data represent the mean +/- SEM of at least three independent experiments. 4G2 absorbance values of TBEV pre-incubated with mabs A5 or B4 were compared to those obtained with the control by using ANOVA and Dunnett’s multiple comparisons test. *, p < 0.05.

The FL at the tip of DII has the capacity to insert into lipid membranes, but at neutral pH it is buried in a pocket lined by residues contributed by DI and DIII of the other subunit in the E dimer (Fig 1A). Under conditions of natural infection, the FL becomes only exposed in the endosome through acidic-pH-induced dissociation of E after viral uptake by receptor-mediated endocytosis [28]. Due to the location of the epitope recognized by mab A5 at the E dimer interface (Fig 1A), we speculated that antibody binding might lead to the dissociation of the dimer and exposure of the FL at neutral pH, allowing for its direct interaction with the plasma membrane. To assess this possibility, we performed chemical cross-linking experiments with a recombinant soluble E (sE) dimer of TBEV in the absence and presence of mab A5. The FL-specific, non-neutralizing mab 4G2 [37, 38] was used as a control. As shown in Fig 6A, cross-linking of sE yielded bands at ~50 kD and ~100 kD corresponding to the monomeric and dimeric form of the protein (Fig 6A, lane 1), in agreement with the dimeric structure of E [39, 40]. In the presence of mab A5, however, E dimers were absent and only bands of E monomers were visible, indicating that the E dimers had dissociated upon complex formation with mab A5. In the case of the control mab 4G2, however, no change of the cross-linking pattern of sE was observed (Fig 6A). Note that the rabbit antiserum used for detection of TBEV E in the blot exhibited some reactivity with the mabs, leading to background bands at Mr around 150 kD. The absence of higher molecular weight bands indicates that cross-linking between E and mabs did not occur under the conditions used.

We further assessed whether binding of mab A5 also led to exposure of the FL in the context of the virus particle. Since cross-linking of whole virions yields a complex pattern of oligomeric bands, meaningful interpretations with respect to mab A5-induced changes would not be possible in experiments similar to those conducted with the sE dimer [41]. Therefore, we studied putative exposure of the FL induced by mab A5 in an ELISA. TBEV was captured with a polyclonal anti-TBEV serum, and biotinylated mab 4G2 was used as a specific detector for the exposed FL [37, 38]. The assay was performed in the absence of detergent to preserve the native particle structure [38, 42]. The data in Fig 6B illustrate that incubation of TBEV with mab A5 resulted in a significantly higher reactivity of mab 4G2 compared to the control without A5. This is in contrast to the non-dissociating DIII-specific mab B4, which did not increase 4G2 binding [40].

### Enhanced cell binding and infectivity is caused by FL interactions

**Fig 7:**
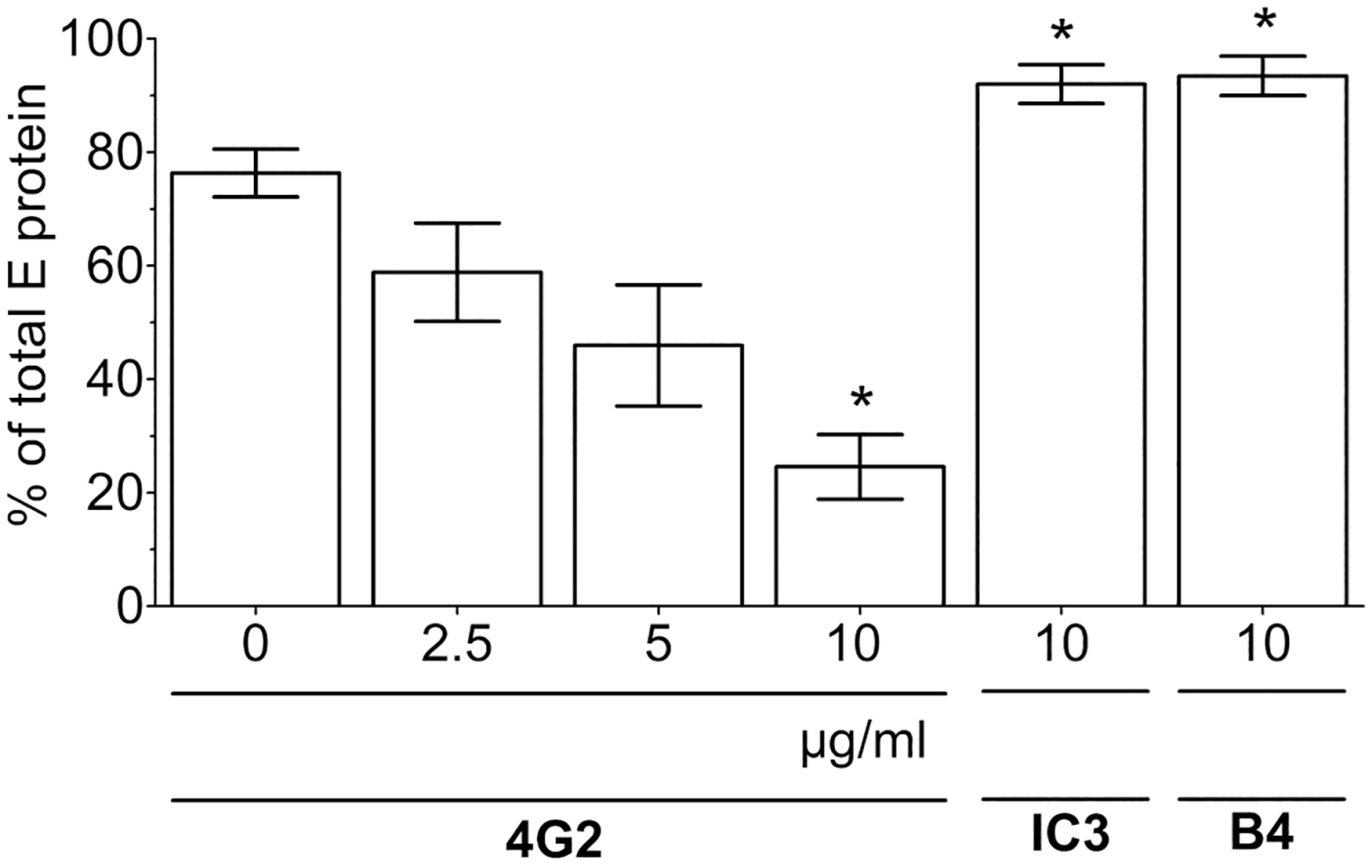
Blocking of mab A5-enhanced binding to liposomes. Mixtures of TBEV with mab A5 were incubated with increasing concentrations of the FL-specific mab 4G2 (0, 2.5, 5, 10 μg/ml) or IC3 (10 μg/ml) or B4 (10 μg/ml) before attachment to liposomes for 20 minutes at 37°C. The y-axis indicates percent bound virus relative to input virus using the amounts of E protein determined by quantitative ELISA. Data represent the mean +/- SEM of at least three independent experiments. The amounts of TBEV bound to liposomes in the presence of different antibodies were compared to those obtained with the TBEV-A5 complex in the absence of these mabs (first column, 0 μg/ml) with ANOVA followed by Dunnett’s multiple comparisons test. *, p < 0.05.

To corroborate that mab A5-induced FL exposure is indeed responsible for enhanced membrane binding, we first analyzed the effect of the FL-specific mab 4G2 on mab A5-induced binding to liposomes. As shown in Fig 7, this activity was inhibited in a concentration-dependent manner by the addition of mab 4G2, but not by mabs directed to other sites (DI—IC3 and DIII—B4). We further verified this finding with another FL-specific mab (A1, [38, 43]) and obtained similar blocking activities (S2 Fig).

**Fig 8:**
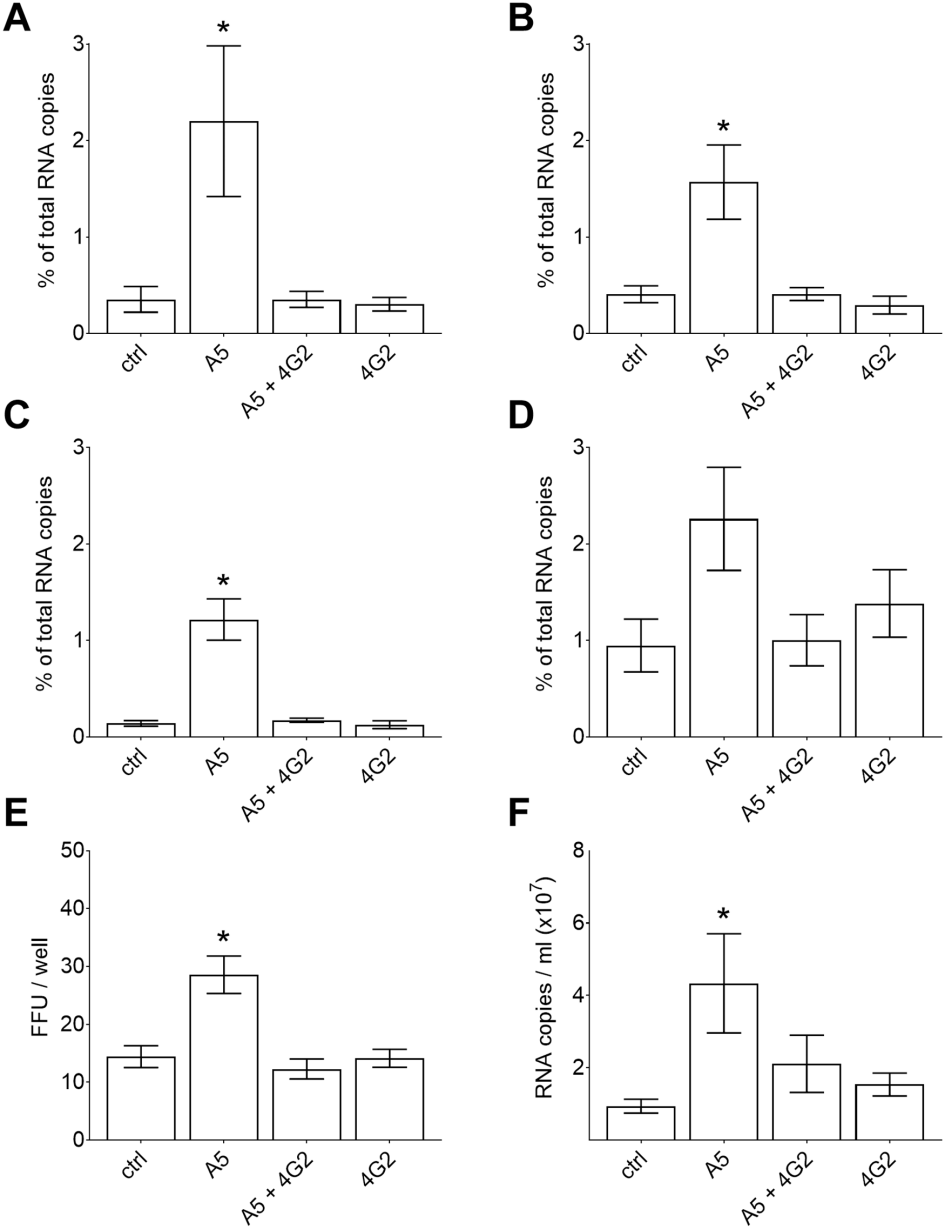
Blocking of mab A5-enhanced cell binding and infectivity by the FL-specific mab 4G2. (A-D) Binding of TBEV to cells after incubation with different mabs (A5, A5+4G2, 4G2) for 1 hour at 4°C. (A) HeLa cells, (B) Vero cells, (C) HFFs, (D) immature moDCs. The y-axis indicates percent bound virus relative to input virus using RNA copy numbers determined by qPCR. (E) Infectivity of TBEV after incubation with different mabs (A5, A5+4G2, 4G2) on HeLa cells using focus formation assays. (F) TBEV production in Vero cells after infection in the presence of different mabs (A5, A5+4G2, 4G2). In contrast to HeLa cells, TBEV did not produce foci in Vero cells. Virus production was therefore measured by quantifying RNA copy numbers with qPCR. Data represent the mean +/- SEM of at least three independent experiments. The amounts of bound TBEV-mab complexes or their infectivity were compared to the corresponding values obtained with the control (ctrl, TBEV without mabs) with ANOVA followed by Dunnett’s multiple comparisons test. *, p < 0.05.

The same effect of mab A5-enhanced binding and its inhibition by mab 4G2 was observed in experiments with HeLa cells, Vero cells, HFFs, and immature moDCs (Fig 8A–8D). These data are in accordance with FL-mediated binding of the virus to the plasma membranes of both primary cells and established cell lines. Although mab 4G2 is non-neutralizing under standard assay conditions, it acquired neutralizing activity when infection of HeLa as well as Vero cells was performed in the presence of mab A5 (Fig 8E and 8F), consistent with the involvement of the FL in the mab A5-mediated infection process.

Since the exposure of the FL can also be influenced by the maturation state of the virus [44], we analyzed the protein composition of the TBEV preparation used in our analyses. As shown in S3 Fig, the TBEV preparation was largely mature, since it did not contain significant amounts of prM, in contrast to an immature control virus preparation obtained by growing the virus in the presence of ammonium chloride, which prevents prM cleavage.

To rule out the possibility that mab A5 binds specifically to the plasma membrane, we tested its interaction with HeLa cells by FACS analyses. As controls, we used mabs IC3, B4 and 4G2 (Fig 1A) and the FcγR-positive K562 cell line. None of the mabs showed any specific binding to HeLa cells (S4 Fig), ruling out that the effects observed were due to direct binding of the mab to the cellular plasma membranes.

Taken together, we conclude that the phenomenon of mab A5-induced enhancement of infection was indeed the result of increased virus attachment to the cells via the exposed FL.

### Mab-A5-mediated enhanced cell binding does not lead to fusion at neutral pH

**Fig 9:**
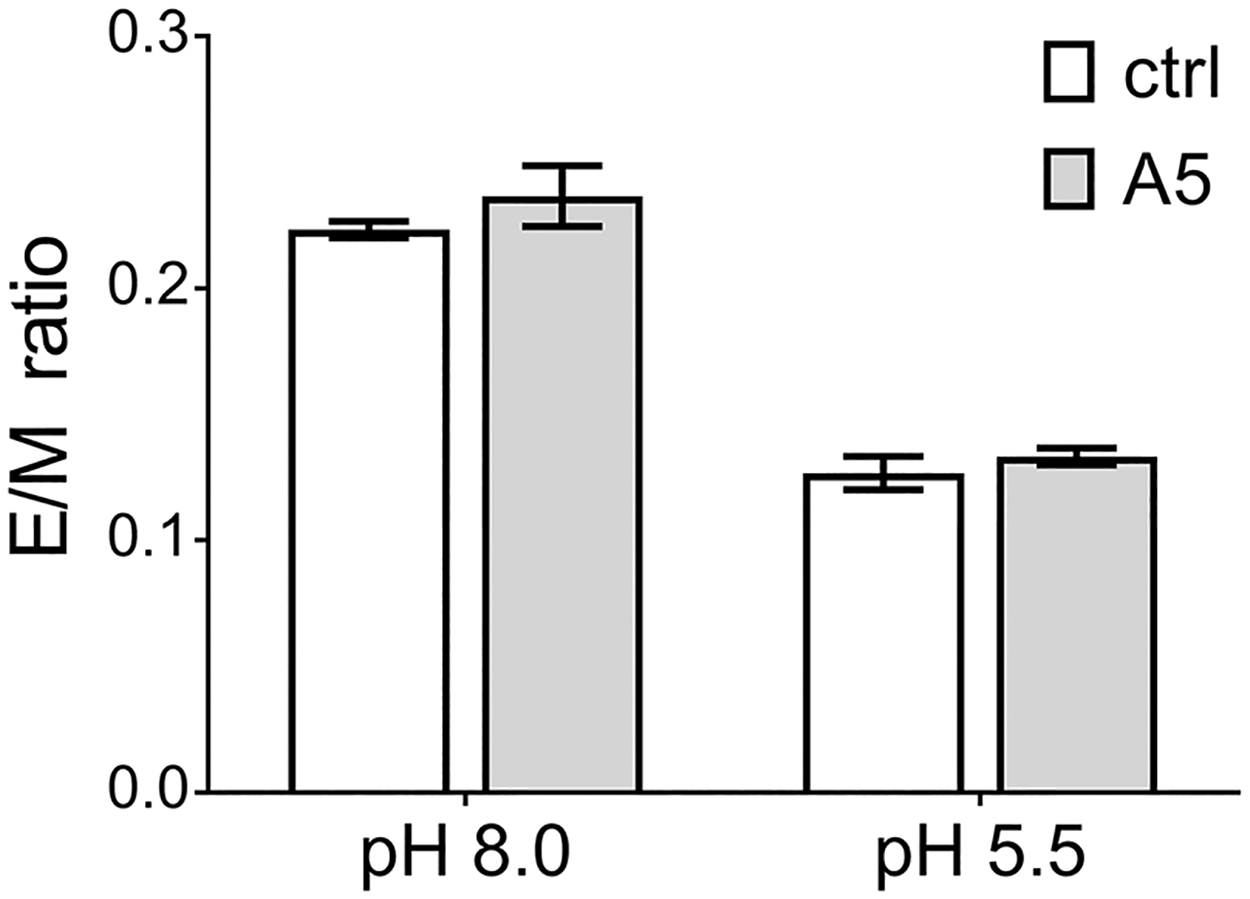
Fusion of pyrene-labeled TBEV with liposomes. Pyrene-labeled TBEV was allowed to interact with liposomes at pH 8.0 or pH 5.5 in the absence or presence of mab A5. The results are expressed as the ratio of pyrene excimer to monomer (E/M), which decreases upon fusion of the viral membrane with the liposomal membrane. Data represent the mean +/- SEM of three independent experiments.

From the previous experiments it was unclear whether the antibody-mediated FL-exposure and membrane attachment might proceed to fusion already at neutral pH. We therefore conducted in vitro fusion assays using liposomes and TBEV labeled with the fluorescent probe pyrene (Materials and methods). In the case of fusion, pyrene-containing phospholipids are diluted into the liposomes, resulting in the decrease of pyrene excimer fluorescence [45, 46]. These experiments were performed in the presence and absence of mab A5, both at neutral and acidic pH. As can be seen in Fig 9, mab A5 was unable to induce fusion at pH 8 and did not inhibit fusion at low pH.

## Discussion

**Fig 10:**
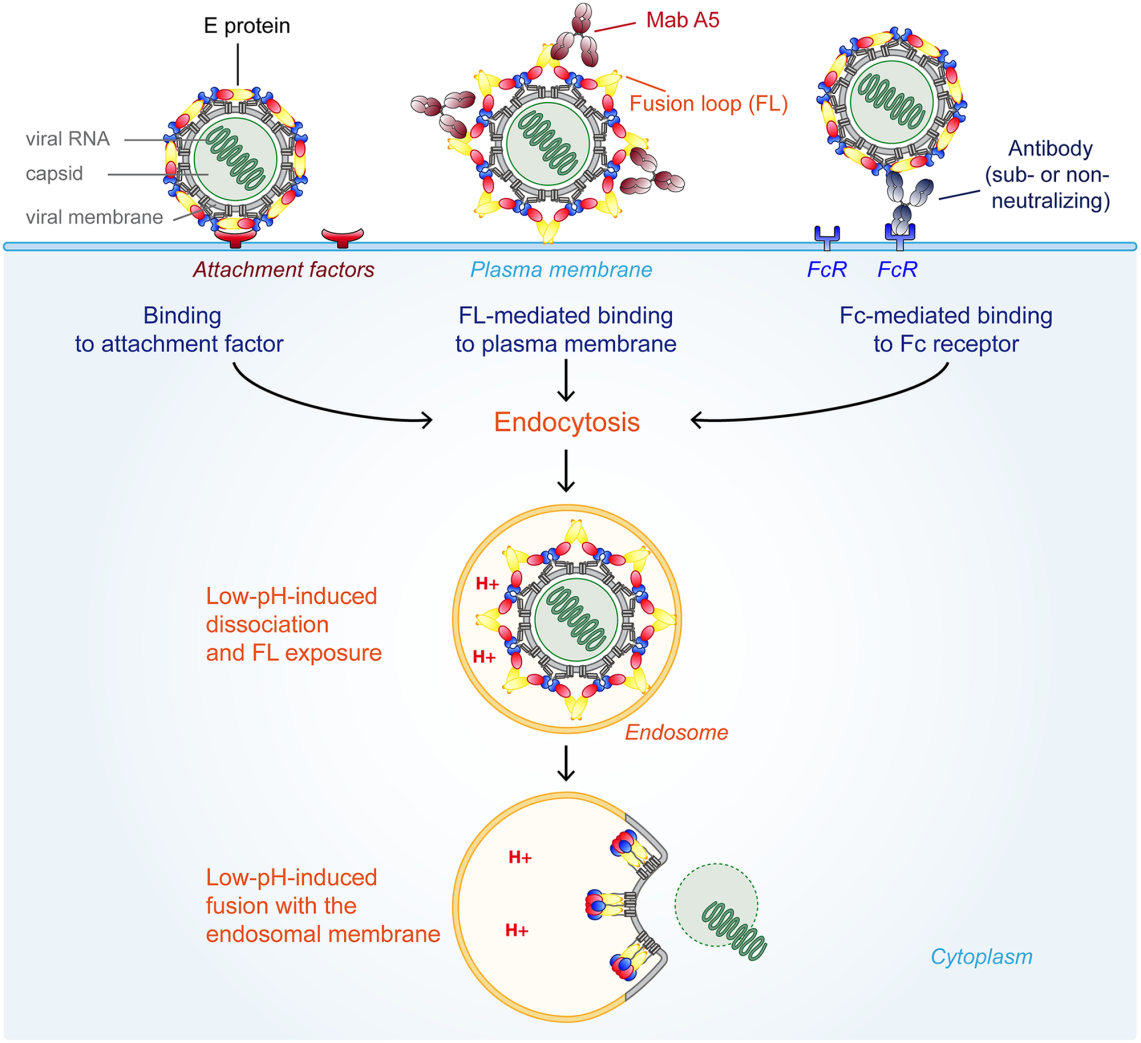
Schematic representation of different modes of virus entry. Three different modes of internalization are shown. Left: after binding to a specific receptor Middle: antibody-induced exposure of the FL and attachment to the plasma membrane Right: FcR-mediated uptake of antibody-virus complexes Color code E: domain I—red, domain II—yellow, domain III—blue, fusion loop—orange, stem-anchor region—gray. For simplification the M protein is not shown in the virus particles.

We have identified a new mechanism by which E protein-specific antibodies can enhance the binding of a flavivirus to the plasma membrane of target cells and thus increase its infectivity. This mechanism differs from the well-described phenomenon of antibody-dependent enhancement of infection, which is mediated by the binding of infectious virus-antibody complexes to FcγRs that are expressed on myeloid cells and trigger virus internalization [18] (Fig 10). In contrast, our experiments provide evidence for a different process of antibody-induced enhancement of infection independent of FcγRs that is mediated by the dissociation of the E dimer (Fig 6A), the exposure of the otherwise buried FL (Fig 6B), and the increased binding of the virus to the plasma membrane of target cells (Figs 4A and 8A–8D). This mechanism appears to be based on the structural dynamics of the flavivirus envelope, antibody-promoted conformational changes, and membrane interactions of the FL that normally occur only in endosomes as a first step of low-pH-triggered viral membrane fusion (Fig 10) [28, 29].

Enhancement of infection of FcγR-negative cells (Vero, BHK-21) was also described in experiments with the domain II-specific mouse mab E100, directed against the E protein of West Nile virus (WNV) [47], and the domain II-specific mouse mabs 1A5D-1 as well as 10A1D-2, directed against the E protein of dengue virus serotype 2 [48]. In addition, in the context of screening for neutralizing activity using Vero cells, cell culture supernatants of some B cells from dengue infected patients also appeared to contain infectivity—enhancing antibodies [49]. The mechanism underlying these observations, however, was not resolved in these studies. Similarly, experiments with Sindbis virus, a member of another family of spherical enveloped viruses with icosahedral structure (alphaviruses [50]), demonstrated that a mouse mab specific for an epitope in the receptor-binding protein E2 (MCAB 49) increased the number of plaque forming units in BHK-21 cells [51]. In this case, increase of specific infectivity was correlated to increased cellular binding of Sindbis virus. The authors hypothesized that their observation might be due to antibody-mediated conformational changes promoting tighter binding of virions to the cell surface. Whether this augmented binding also involved the FL (present in the alphavirus fusion protein E1) remains to be elucidated. Interestingly, enhanced infection was only seen with a specific strain of Sindbis virus but not with others, which may be related to differences in viral envelope dynamics, similar to those described for flaviviruses [52–54].

An apparently different mechanism of enhanced infection of FcγR-negative cells from humans (A549), hamsters (BHK-21), mice (NIH3T3), and mosquitoes (C6-36) was demonstrated in experiments with dengue 2 virus (strain PL046) and a mab specific for the prM protein [55], which forms a complex with E in immature forms of flaviviruses [8]. These findings were discussed to be caused by molecular mimicry of prM of a cellular protein (most likely HSP60), allowing the simultaneous binding of the bivalent antibody to the virus as well as to the cell and thus to increase cellular attachment. This contrasts to the mechanism of enhancement described here. The combination of mab A5 with TBEV appears to be independent of interactions with cellular receptor-like proteins, because it can also be demonstrated with plain liposomes (Fig 4B), suggesting that it is mediated by FL-interactions with the lipids in target membranes.

Indirect evidence for antibody-promoted structural changes that increase the exposure of the FL had already become apparent in competitive binding studies with E-specific mabs [56, 57]. Studies with TBE and dengue viruses provided evidence that the binding of antibodies to epitopes in domains II and III of E could lead to increased binding of FL-specific mabs. Detailed structural information about influences of antibody binding on the envelope protein arrangement of a flavivirus was first obtained by Lok et al. [25] using cryo EM of dengue 2 virus (strain NGC) in complex with a mab to the A strand of DIII. As discussed by Cockburn and colleagues [27], such effects may also be caused by ligands other than antibodies and might contribute to the tuning of infection as well as cellular tropism. Beyond that, the exposure of otherwise cryptic protein surfaces by antibody binding can also modulate the fine specificity of antibody responses upon immunization with immune complexes [40].

The binding of the infection-enhancing mab A5 to the E dimer appears to interfere with the equilibrium between monomeric and dimeric states, favoring the monomer and exposing the FL. The epitope of A5 involves residues in DII lying at the interface of the monomers in the E dimer as revealed by the analysis of neutralization escape variants [58] and mutational mapping [59]. Interestingly, FcγR-independent enhancement of WNV infection was observed with a mab (E100) that, like mab A5, binds to an epitope at a similar site in domain II and the E dimer interface [47]. Based on this similarity, the mechanism of increased infectivity may be analogous and involve antibody-promoted E dimer dissociation in both virus systems.

Since both mab A5 and mab E100 augment rather than inhibit infection, one has to assume that they are unable to block fusion in the endosome, even when they are co-internalized with the virus. Post-entry neutralization (reviewed in [60]) would require maintenance of the virus-antibody interaction at the acidic pH in endosomes. In this context, it is of note, that the epitopes of both mab A5 and mab E100 involve histidines, H208 in TBEV and H263 in WNV [47]. It is quite possible that protonation of these residues in the endosome might weaken or even abolish the antibody interactions with E. Domain and oligomeric rearrangements required for membrane fusion [28] would thus not be inhibited by antibodies specific for E, allowing for infection to proceed unimpaired.

The FL-mediated binding to cellular plasma membranes—through E dimer dissociation induced by mab A5—mimics the first step of the viral fusion process that is normally triggered by the acidic pH in endosomes [29]. One could therefore speculate that fusion would occur already at this stage of antibody-mediated infection. However, we did not detect any mab-A5-induced fusion activity at neutral pH using liposomes (Fig 9) similar to what was observed upon E dimer dissociation and FL exposure at alkaline pH [41]. These negative results are consistent with structural considerations that argue against the possibility of fusion without acidification, because the relocation of DIII—required for the fusogenic conformational change of E [28]—appears to be dependent on the protonation of one or more histidines at the interface of domain I and III [61]. Taken together, all the above data strongly suggest that mab A5-induced binding to the plasma membrane facilitated virus uptake by endocytosis.

A striking observation of our binding experiments was the low degree of TBEV attachment to both transformed cell lines and primary cells under normal conditions of infection (Fig 2). Dengue virus bound with similar low efficiency to HeLa cells but—in contrast to TBEV—efficiently to immature dendritic cells, which express DC-SIGN, a previously identified attachment factor for dengue viruses (reviewed in reference [62]). The difference appears to be due to interaction of E with DC-SIGN via a glycan at N67 [12], which is present in dengue viruses but absent in TBEV and other flaviviruses [63]. A very low efficiency of cellular attachment is apparently sufficient for infection. Similar results (less than 1% binding of input virus) were obtained by Van der Schaar et al. [64] with the dengue 2 virus strain PR159S1 and monkey kidney cells (BS-C-1). The low efficiency of binding to certain cells may be responsible for the low specific infectivity of flaviviruses observed in many instances [14, 64]. Serial transfer experiments of dengue virus [64] and TBEV (Fig 3) indicate that conventional infectivity determinations lead to a gross underestimation of the real number of infectious particles present in virus suspensions, because of the low degree of virus binding to cells under the conditions of these assays.

The identification of a novel mechanism of antibody-enhanced infection adds an additional facet to the complexity of virus interactions with the variable mixtures of antibodies in polyclonal sera. Influenced by various factors such as binding sites and avidities, the effects of antibody populations on the virus can be expected to be additive, cooperative or competitive and thus modulate neutralizing and infection-enhancing activities in different ways, depending on the antibody compositions of such sera. Importantly, the antibody composition can vary strongly among individuals [65–67] and is especially influenced by anamnestic responses as a result of the history of previous infections and vaccinations [20, 40, 68–70]. In addition to the heterogeneity of antibody responses, FL-mediated enhancement effects can also be influenced by properties of the virus (breathing dynamics and extent of maturation, reviewed in references [22, 71]). Future studies beyond the proof-of-principle presented here will be necessary to dissect this complexity and investigate whether and to which extent antibody-mediated enhancement in FcγR-negative cells can have implications for immunity and pathogenesis of human flavivirus infections.

## Materials and methods

### Ethics statement

Primary human foreskin fibroblasts (HFFs) were cultivated from anonymous dissociated foreskin samples (Department of Pediatric Surgery, Medical University of Vienna, Vienna, Austria) and used for virus interaction studies according to the ethical approval from the ethics committee of the Medical University of Vienna (ECS 2061/2012).

Chicken embryo cells were prepared from 10-day-old embryonated eggs (VALO Biomedia GmbH, Germany). Chicken embryos within the first two thirds of their development do not fall under the “Austrian Law on the Protection of Animals Used for Scientific Purposes” (“Gesamte Rechtsvorschrift für Tierversuchsgesetz”), and therefore approval by an animal ethics committee was not required.

Human serum samples from confirmed TBEV infections were originally sent to the Center for Virology for diagnostic purposes and used anonymously with the approval of the local ethics committee of the Medical University of Vienna (EK 134/2008).

### Cell lines

HeLa cells (HeLa-H1, strain Ohio, ATCC) were cultured in Dulbecco’s Modified Eagle Medium (DMEM, Gibco) supplemented with 10% fetal calf serum (FCS, Gibco). Primary human fibroblasts (HFFs) were grown in Minimum Essential Medium Eagle (MEM, Sigma-Aldrich) supplemented with 10% FCS and 1% non-essential amino acids solution (Gibco). Vero cells (ECACC) were grown in DMEM (Gibco) supplemented with 5% FCS (Gibco). K562 cells (ATCC) were grown in Iscove's Modified Dulbecco's Medium (IMDM, Gibco) supplemented with 10% FCS (Gibco).

### Monocyte-derived dendritic cells (moDCs)

Immature moDCs were prepared from whole blood of anonymous donors, purchased from the Austrian Red Cross Blood Bank (Vienna, Austria).

Peripheral blood was diluted 1:1 with phosphate-buffered saline pH 7.4 (PBS, Sigma-Aldrich) and mononuclear cells were separated using Ficoll-Paque (GE Healthcare Life Science). CD14+ cells were obtained by MACS (magnetic cell isolation and preparation) using anti-CD14 magnetic beads according to the manufacturer’s protocol (Miltenyi Biotech). The purity of the cells was ≥ 95%, as determined by flow cytometry analysis using a mouse anti-human CD14 antibody (BD Bioscience) (S1 Fig).

To generate immature dendritic cells, 1x10^6^ purified CD14+ cells were cultured in RPMI-1640 medium (Sigma-Aldrich), supplemented with 10% FCS, recombinant human GM-CSF (100 ng/ml, PeproTech) and recombinant human IL-4 (25 ng/ml, PeproTech) for 5 days [72]. On day 5 (prior to binding assays), phenotypic characterization of the cells was carried out by flow cytometry analysis using conjugated mouse anti-human antibodies (BD Bioscience) directed to the surface molecules CD209, CD1a, HLA-DR, CD80, CD83 and CD86. As shown in S1 Fig, ~90% of the cells were CD209 (DC-SIGN) and CD1a positive. The cell population had a typical immature moDC profile with high expression of CD209, CD1a as well as HLA-DR and low (or intermediate) expression of CD80, CD83 and CD86 (S1 Fig) [73, 74].

### Virus preparations

#### TBEV

Primary chicken embryo cells were infected with TBEV strain Neudoerfl (GenBank accession no. U27495). Virus-containing cell culture supernatants were harvested 24–48 hours after infection, concentrated by ultracentrifugation and purified by rate-zonal centrifugation [75]. BSA (0.1% final concentration, Serva) was added to the virus-containing fractions and aliquots were frozen at -80°C. For liposome binding assays and detection by ELISA, the virus was further purified by equilibrium sucrose density gradient centrifugation [75]. The maturation state of the virus preparation was analyzed by sodium dodecyl sulfate (SDS)–15% polyacrylamide gel electrophoresis (PAGE) and Coomassie staining (S3 Fig). As a control, an immature virus preparation was included which was obtained by growing TBEV in the presence of 20 mM NH_4_Cl and purification by rate as well as equilibrium centrifugation, as described previously [76].

For membrane fusion assays, virions were metabolically labeled with 1-pyrenehexadecanoic acid as described previously [45].

#### RV-A2

HeLa cells were infected with RV-A2 at an MOI of 1 and incubated for 18–24 hours at 34°C. Virus-containing cell culture supernatants were harvested, clarified by centrifugation and filtered (0.2 μm pore size, Sarstedt). Aliquots were stored at -80°C.

#### Dengue virus

Vero cells were infected with the DENV2 strain NGC in medium 199 (Sigma-Aldrich), containing 15mM HEPES, 15mM HEPPS, and 0.1% BSA. 48 to 72 hours after infection virus-containing cell culture supernatants were harvested and clarified by centrifugation. Aliquots were stored at -80°C.

### Production and purification of mabs and sE

Mabs A1, A5, B4, IC3, and 4G2 [77–80] were purified from serum-free hybridoma cell culture supernatants (described in references [77–79]) using protein A or G Sepharose High Performance columns according to the manufacturer’s instructions (GE Healthcare Life Sciences). The hybridoma cell line HB 112: D1-4G2-4-15 (producing mab 4G2) was obtained from ATCC. Specific characteristics of the mabs used in this study are summarized in Table 1.

**Table 1:** Characteristics of mabs used in this study.

Mab	Isotype	Immunogen	Epitope location	References
A5	IgG1	TBEV E protein complexes (rosettes)	DII	[58, 78]
B4	IgG1	TBEV E protein complexes (rosettes)	DIII	[58, 78]
IC3	IgG2b	Formalin-inactivated TBEV	DI	[79, 81]
A1	IgG2	TBEV E protein complexes (rosettes)	FL (DII)	[43, 78]
4G2	IgG2a	Infectious dengue virus serotype 2	FL (DII)	[37, 80]

Recombinant TBEV sE (strain Neudoerfl, amino acid 1 to 400) containing a strep-tag was expressed in Drosophila S2 cells (Invitrogen) and purified by StrepTactin affinity chromatography as described previously [40, 66].

The purity and size of the mabs as well as sE were controlled by Agilent 2100 Bioanalyzer electrophoresis and/or SDS-PAGE (15% polyacrylamide gels). The verification of the oligomeric structure and correct folding of the recombinant sE protein was described in a previous study [66].

### Measuring the effect of antibodies on virus infectivity in focus assays

Fifteen to 20 focus forming units (FFU) of TBEV were pre-incubated with serial dilutions of mabs for 1 hour at RT. TBEV-antibody complexes were then added to pre-chilled confluent HeLa cell monolayers in Medium199 (Sigma-Aldrich) containing 15 mM HEPES, 15 mM HEPPS, 0.1% BSA. After 1 hour at 4°C, the inoculum was removed and cells were washed twice to remove unbound virus. DMEM (1% FCS) was added and the temperature was shifted to 37°C to allow infection of the cells. After 30 minutes incubation at 37°C, the cells were overlaid with 3% carboxymethyl cellulose in DMEM (1% FCS). Two days after infection, the cells were fixed with 4% paraformaldehyde for 20 minutes at RT and permeabilized for 30 minutes at 37°C with a Tris buffer (50 mM Tris, 150 mM NaCl, pH 7.6) containing 3% nonfat dry milk powder, 0.5% Triton X-100, and 0.05% Tween 20. Foci were visualized with a virus-specific polyclonal rabbit anti-TBEV serum (obtained from the Core Unit of Biomedical Research, Division of Laboratory Animal Science and Genetics, Medical University of Vienna, Himberg, Austria), alkaline phosphatase-labeled goat anti-rabbit immunoglobulin G (Sigma-Aldrich) and SigmaFast Fast Red TR/naphthol AS-MX (Sigma-Aldrich) as a substrate.

For the inhibition of mab A5-induced enhancement of infectivity, TBEV-mab complexes (15–20 FFU TBEV, final mab concentration 5 μg/ml) were further incubated with mab 4G2 or control mabs (final concentration for each mab 5 μg/ml) for 1 hour at RT.

For serial transfer experiments, TBEV was added to pre-chilled HeLa cell monolayers, incubated for 30 minutes at 4°C, harvested and then transferred onto fresh cells. To permit infection of bound virions, the cells were washed, pre-warmed medium was added, and the cells were shifted to 37°C. After 30 minutes at 37°C, the cells were covered with an overlay and incubated for 2 days at 37°C. The procedure was repeated nine times and foci were visualized as described above.

### Measuring virus growth

TBEV (1x10^9^ RNA copies) with and without mabs (20 μg/ml) was pre-incubated for 1 hour at RT. As described for the focus assays, the virus-antibody complexes were added to confluent HeLa or Vero cell monolayers and incubated for 1 hour at 4°C. The cells were washed, fresh medium was added, and the temperature was shifted to 37°C for infection. Eight, 16 and 24 hours post infection aliquots were taken from the HeLa cell culture supernatant and the infectivity titers were determined in focus assays.

In the case of Vero cells, the cell culture supernatant was harvested 24 hours after infection and the number of RNA copies was determined by qPCR.

### Biotinylated liposomes

L-α-phosphatidylcholine (25 mol%), L-α-phosphatidylethanolamine (22.5 mol%), 1,2-dipalmitoyl-sn-glycero-3-phosphoethanolamine-N-(cap biotinyl) (1.25 mol%), 1,2-dioleoyl-sn-glycero-3-phosphoethanolamine-N-(cap biotinyl) (1.25 mol%, all from Avanti Polar Lipids), and cholesterol (50 mol%, Sigma- Aldrich) [82, 83] in chloroform were dried to a thin film with a rotary evaporator in a high vacuum as described previously [82]. The lipid film was hydrated in liposome buffer (10 mM triethanolamine, 140 mM NaCl, pH 8.0) and subjected to 5 cycles of freezing and thawing. Liposomes were extruded through two 200 nm polycarbonate membranes by the use of a Liposofast syringe type extruder (Avestin) [82].

### Liposome binding assay

TBEV (0.5 μg/ml E protein) was pre-incubated with and without mabs (20 μg/ml) for 1 hour at room temperature (RT). Then, biotinylated liposomes were added to a final concentration of 0.2 mM for 20 minutes at 37°C. The liposomes were aggregated by cross-linking with avidin (Sigma-Aldrich) and pelleted at 1,000 g for 15 minutes at RT. The pellet was resuspended in TAN buffer (50 mM triethanolamine, 100 mM NaCl, pH 8.0) containing 0.5% Triton X-100. The amount of E protein in the pellet (bound) as well as in the supernatant (unbound) was quantified by 4-layer ELISA [76].

For the inhibition of mab A5-induced enhancement of binding, TBEV-mab complexes (0.5 μg/ml E protein, final mab concentration 10 μg/ml) were further incubated with serial dilutions of mab 4G2 (2.5, 5, 10 μg/ml), mab A1 (2.5, 5, 10 μg/ml) or control mabs (final concentration for each mab 10 μg/ml) for 1 hour at RT.

### Cell binding assay

Virus (1x10^9^ RNA copies of TBEV, DENV or RV-A2) was pre-incubated with and without mabs (20 μg/ml) for 1 hour at RT. HeLa cells, Vero cells and HFFs were detached with the non-enzymatic EDTA versene solution (Gibco). 2x10^5^ HeLa cells or 2x10^5^ Vero cells or 2x10^5^ HFFs or 1x10^6^ immature moDCs were added at 4°C, 30°C or 37°C in binding buffer (medium 199 (Sigma-Aldrich), containing 15 mM HEPES, 15 mM HEPPS, 0.1% BSA, pH 7.6). Twenty mM NH_4_Cl was included to prevent fusion in endosomes, release of the genome and its replication. After incubation for 1 hour at the respective temperature, cells were pelleted by low-speed centrifugation and washed twice with ice-cold PBS pH 7.4 (Sigma-Aldrich) to remove unbound virus. The viral RNA was extracted from the pellet and supernatant using RNeasy Mini kit (Qiagen) according to the manufacturer’s protocol. The number of bound and unbound genomic RNA copies was quantified by two-step quantitative reverse transcription PCR (qPCR).

To block the engagement of FcγRs by TBEV in the presence of virus-specific mabs, 1x10^6^ HeLa cells or 1x10^6^ FcγR-positive K562 were pre-treated with 2.5 μg of the mouse anti-human Fcγ RII/CD32a monoclonal antibody (R&D Systems) for 90 minutes at 37°C, as described in reference [35]. The pre-treated cells were then washed with medium and used for cell binding experiments.

### Fusion assay (lipid mixing assay)

Fusion of virions with liposomes was determined by a change in the pyrene monomer and excimer fluorescence caused by the dilution of pyrene-labeled viral phospholipids into the unlabeled liposomal membrane [45, 46]. TBEV with and without mab A5 (same molar ratios as used for the liposome binding assays) was pre-incubated for 1 hour at room temperature (RT). Liposomes were added and the mixture was either treated with TAN buffer pH 8.0 or with 450 mM 2-(N-morpholino)ethanesulfonic acid (MES), pre-titrated to yield a final pH of 5.5 in the assay. After an incubation for 1 hour at 37°C, the samples were back-neutralized with 300 mM triethanolamine. Fluorescence emission spectra were recorded at 21°C from 360 to 520 nm using an excitation wavelength of 343 nm and a Perkin Elmer LS 50B Fluorescence Spectrophotometer. The excimer and monomer peaks were measured at emission wavelengths of 480 and 397 nm, respectively.

### Quantitative four-layer ELISA

E protein was quantified by a 4-layer enzyme-linked immunosorbent assay (ELISA) as described previously [76]. The standard, purified TBEV, and the samples were denatured with 0.4% SDS for 30 minutes at 65°C [76].

### Quantitative PCR

cDNA was synthesized from isolated viral RNA with the iScript cDNA Synthesis Kit (Bio-Rad). For the two-step quantitative reverse transcription PCR (qPCR), cDNA was mixed with the respective probes (10 pmol, VBC biotech) and primers (25 pmol, VBC Biotech), and TaqMan Universal PCR Mastermix (LifeTechnologies).

The following primers and probes were used:

TBEV—forward primer: GAAGCGGAGGCTGAACAACT, reverse primer: TTGTCACGTTCCGTCTCCAG, probe: 5’-FAM-TGTGTACAGGCGCACCGGCA-TAMRA-3’.

DENV2—forward primer: CAGATGGAGGGAGAAGGAGTC, reverse primer: CGCCCTACTCTTGCTAACCA, probe: 5’-FAM-ACAGTCACAGAAGAAATCGCCGTGCA-TAMRA-3’. RV-A2—forward primer: GGCCCCTGAATGTGGCTAA, reverse primer: AAGTAGTTGGTCCCATCCCG, probe: 5’-FAM-CCCTGCAGCTAGAGCACGTAACCC-TAMRA-3’.

The temperature profile of the reaction was 3 minutes at 50°C, 10 minutes at 95°C, followed by 45 cycles of 15 seconds at 95°C, 30 seconds at 55°C, and 30 seconds at 72°C [84, 85]. Serial ten-fold dilutions of the plasmids pTNd/c TBEV (containing the full-length genomic cDNA insert of TBEV strain Neudoerfl, [86]), plasmid bluescript HRV2 (containing the full-length genomic cDNA insert of RV-A2, [87]) and plasmid MA-T DENV2 (containing a part of the NS5 sequence of DENV2 NGC; synthesized by Invitrogen) were used to generate a standard curve for quantification, as described previously for DENV2 [64] and TBEV [88].

### Chemical cross-linking analysis, SDS PAGE and Western blotting

TBEV sE (1.5 μg) and mabs (4 μg) were diluted in 800 μl TAN buffer pH 8.0 and incubated for 1 hour at RT. Chemical cross-linking with 10 mM dimethyl suberimidate (DMS, Pierce) was performed essentially as described previously [40, 89]. The reaction was stopped by the addition of ethanolamine to a final concentration of 10 mM. The samples were precipitated with trichloroacetic acid (TCA), subjected to SDS-PAGE under non-reducing conditions using 5% polyacrylamide gels and a phosphate-buffered system [90]. The proteins were blotted onto polyvinylidene difluoride membranes (Bio-Rad) with a Bio-Rad Trans-Blot semidry transfer cell [91]. Detection was carried out immunoenzymatically using a polyclonal rabbit anti-TBEV serum (obtained from the Core Unit of Biomedical Research, Division of Laboratory Animal Science and Genetics, Medical University of Vienna, Himberg, Austria) together with a horseradish peroxidase (HRP)-linked anti rabbit IgG (from donkey, GE Healthcare Life Sciences) as described previously [91].

### FL exposure ELISA

Microtiter plates were coated with a polyclonal guinea pig anti-TBEV serum (obtained from the Core Unit of Biomedical Research, Division of Laboratory Animal Science and Genetics, Medical University of Vienna, Himberg, Austria). After blocking with 1% BSA in PBS pH 7.4, native TBE virions at an E protein concentration of 1 μg/ml in PBS pH 7.4, containing 2% lamb serum (PAA), were added and incubated for 1 hour at 37°C. No detergent was used at any stage to avoid destabilization of the virion [38, 42]. Mabs (10 μg/ml) were added to the bound virus and incubated for 1 hour at 37°C. Mab-induced FL exposure was analyzed with the biotin-labeled mab 4G2 at a concentration of 1 μg/ml and Streptavidin-HRP (Sigma-Aldrich).

### Blocking ELISA with mab A5 and polyclonal serum samples

Human serum samples were diluted 1:25 in PBS pH 7.4 (containing 2% lamb serum (PAA)) and added to strep-tagged recombinant TBEV sE (25 ng/well) captured on Streptactin-coated plates (IBA GmbH). After incubation for 1 hour at 37°C, the plates were washed with PBS and mab A5 was added (12.5 ng/well). Mab A5 bound to the antigen was detected using peroxidase-labeled rabbit anti mouse IgG (Pierce). Results were expressed as percent mab reactivity (absorbance) in the absence of blocking antibodies.

### Quantification of TBEV-specific IgG antibodies

Quantitative ELISAs with human serum samples were performed as previously described using non-treated microtiter plates coated with 25 ng/well formalin-inactivated TBEV [92]. Dilution series of the human serum samples were added, and the plates were incubated for one hour at 37°C. Bound antibodies were detected with biotin-labeled goat anti-human IgG (Pierce) followed by Streptavidin—Peroxidase (Sigma). TBEV-specific antibodies were quantified in IgG units by using a human post-infection anti-TBEV serum as a standard that was arbitrarily set to contain 1000 units. Standard curves (two-fold dilutions, 7 data points) were fitted with a four-parameter logistic regression (GraphPad Prism 6.0, GraphPad Software Inc.). The cut-off (positive ≥ 220 units) was based on testing 90 flavivirus-negative serum samples [92].

### FACS analyses of HeLa and K562 cells

5x10^5^ HeLa or 5x10^5^ K562 cells were incubated with mabs A5, IC3, 4G2 and B4 (2 μg/ml) for 1 hour at 4°C. Alexa Fluor 488 goat-anti mouse IgG (H+L), cross-adsorbed (Invitrogen), was used for the detection of cell-bound mabs by FACS.

### Statistical analyses

ANOVA followed by Dunnett’s multiple comparison test and Student’s t-test were performed with log-transformed data. GraphPad Prism 6.0 was used for the analyses and p values < 0.05 were considered significant.

## Supporting information

S1 Table TBEV-specific IgG concentrations and mab A5 blocking activity of polyclonal TBEV post-infection sera. TBEV IgG units were determined using a polyclonal human post-infection anti-TBEV serum (standard) set at 1000 units (Materials and methods). Mab A5 blocking activity was analyzed with a competition ELISA as described in Materials and Methods.
(DOCX)

S1 Fig Generation and phenotypic characterization of immature monocyte-derived dendritic cells (moDCs) by flow cytometry. CD14+ cells were isolated from whole blood and differentiated into immature moDCs as described in Materials and Methods. After 5 days, immature moDCs were harvested, characterized by flow cytometry using mabs directed to cell-surface markers and used for binding assays. (A) Representative dot plot of isolated CD14+ cells with side scatter (SSC) versus a FITC-conjugated CD14 mab. Cells were gated for live cells by forward scatter (FSC) and SSC. Purity of isolated CD14+ cells was generally ≥ 95%, in this example >99%. (B) Representative dot plot of immature moDCs with a PE-conjugated CD1a mab versus a FITC-conjugated CD209 (DC-SIGN) mab. Cells were gated for live cells by FSC and SSC. Approximately 90% of the cells were positive for CD1a and CD209. (C) Representative histograms of immature moDCs using the following mabs: APC/H7-conjugated HLA-DR antibody, PE-conjugated CD1a antibody, FITC-conjugated CD209 antibody, APC-conjugated CD83 antibody, PE/Cy7-conjugated CD80 antibody and PerCP/Cy5.5-conjugated CD86 antibody. Immature moDCs typically expressed high amounts of CD1a, CD209 as well as HLA-DR and low or intermediated amounts of CD80, CD83 as well as CD86. Red lines—unstained cells, green lines—stained cells.
(TIF)

S2 Fig Blocking of mab A5-enhanced binding to liposomes by the FL-specific mab A1. Mixtures of TBEV with mab A5 were incubated with increasing concentrations of the FL-specific mab A1 (0, 2.5, 5, 10 μg/ml) before incubation with liposomes for 1 hour at 37°C. The y-axis indicates percent bound virus relative to input virus using the amounts of E protein determined by quantitative ELISA. Data represent the mean +/- SEM of three independent experiments. The amounts of TBEV bound to liposomes in the presence of antibodies were compared to those obtained with the TBEV-A5 complex in the absence of these mabs (first column, 0 μg/ml) with ANOVA followed by Dunnett’s multiple comparisons test. *, p < 0.05.
(TIF)

S3 Fig Maturation state of TBEV. An aliquot of the purified TBEV preparation used in the present work (lane 1) was precipitated with trichloroacetic acid, subjected to SDS-PAGE and stained with Coomassie blue. A purified immature virus preparation obtained by growth in the presence of ammonium chloride was used as a control (lane 2).
(TIF)

S4 Fig Binding of mabs to HeLa cells. (A) Representative histograms of HeLa cells and mabs A5, IC3, 4G2 and B4. (B) Representative histograms of K562 cells and mabs A5, IC3, 4G2 and B4. Cells were gated for live cells by FSC and SSC. Red lines—unstained cells, green lines—cells stained with the Alexa Fluor 488-labeled anti-mouse conjugate, blue lines—cells stained with the mabs and the Alexa Fluor 488-labeled anti-mouse conjugate.
(TIF)
