# Supplementary material for: A novel mechanism of antibody-mediated enhancement of flavivirus infection
Source: PLoS Pathog. 2017 Sep 15;13(9):e1006643. doi: 10.1371/journal.ppat.1006643 (PMC5617232; doi:10.1371/journal.ppat.1006643)
Supplement: S1 Table — TBEV IgG units were determined using a polyclonal human post-infection anti-TBEV serum (standard) set at 1000 units (Materials and methods). Mab A5 blocking activity was analyzed with a competition ELISA as described in Materials and Methods. (DOCX) [file ppat.1006643.s001.docx]

**S1 Table. TBEV-specific IgG concentrations and mab A5 blocking activity of polyclonal TBEV post-infection sera.**

| TBEV sera  No. | TBEV-specific  IgG units^a^ | mab A5 blocking activity (%)^b^  (mean +/- SEM) |
| --- | --- | --- |
| 1 | 13,766 | - |
| 2 | 1,302 | - |
| 3 | 8,245 | - |
| 4 | 9,503 | 54.1% (+/- 1.9%) |
| 5 | 3,375 | - |
| 6 | 8,812 | - |
| 7 | 2,562 | - |
| 8 | 8,203 | - |
| 9 | 11,550 | - |
| 10 | 5,438 | - |
| 11 | 7,763 | - |
| 12 | 18,787 | - |
| 13 | 4,679 | - |
| 14 | 10,560 | - |
| 15 | 9,881 | - |
| 16 | 2,209 | - |
| 17 | 3,494 | - |
| 18 | 3,999 | - |
| 19 | 2,002 | - |
| 20 | 3,477 | - |
| 21 | 5,089 | - |
| 22 | 11,391 | - |
| 23 | 5,007 | - |
| 24 | 1,835 | - |
| 25 | 12,953 | - |
| 26 | 3,704 | - |
| 27 | 5,714 | - |
| 28 | 6,768 | - |
| 29 | 6,496 | - |
| 30 | 10,304 | - |

^a^ TBEV IgG Units were determined using a polyclonal human post-infection anti-TBEV serum (standard) set at 1000 units (Material and Methods).

^b^ Mab A5 blocking activity was analyzed with a competition ELISA as described in Materials and Methods.
