# Supplementary figures and images for: A novel mechanism of antibody-mediated enhancement of flavivirus infection
Source: PLoS Pathog. 2017 Sep 15;13(9):e1006643. doi: 10.1371/journal.ppat.1006643 (PMC5617232; doi:10.1371/journal.ppat.1006643)

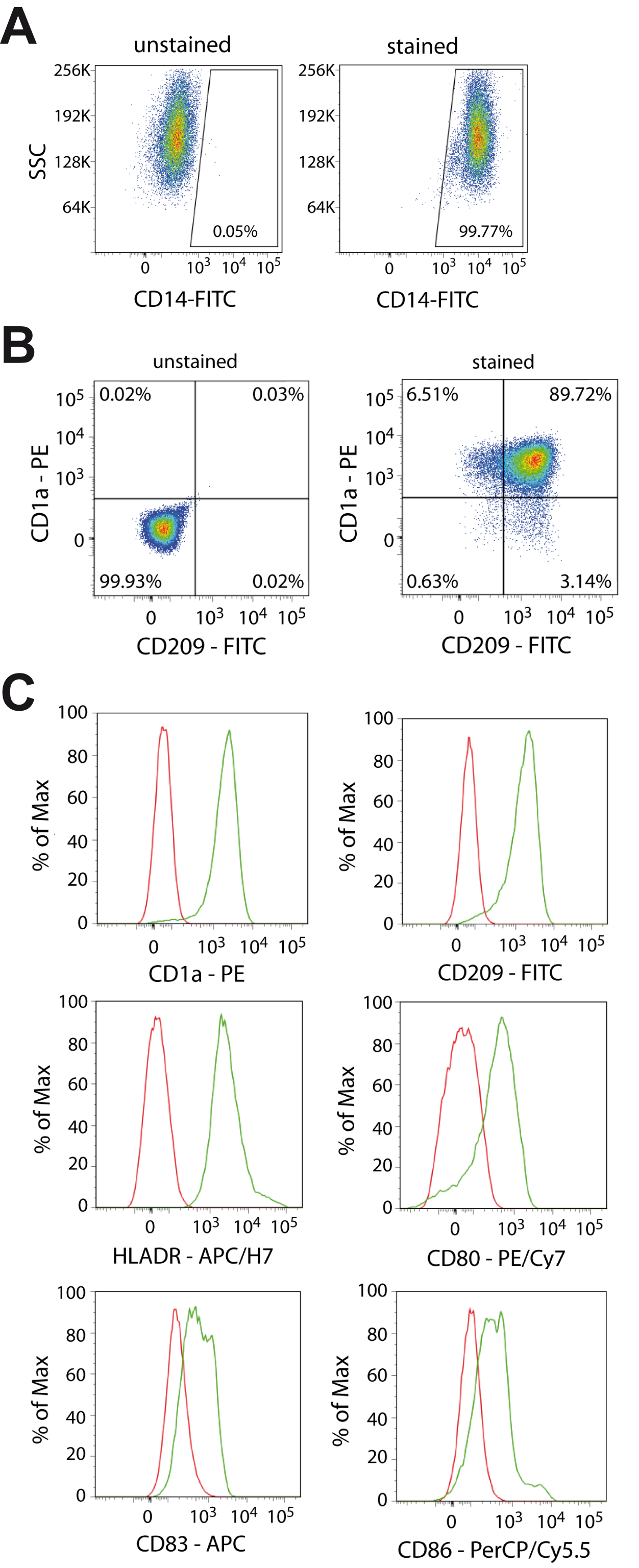

Supplement: S1 Fig — CD14+ cells were isolated from whole blood and differentiated into immature moDCs as described in Materials and Methods. After 5 days, immature moDCs were harvested, characterized by flow cytometry using mabs directed to cell-surface markers and used for binding assays. (A) Representative dot plot of isolated CD14+ cells with side scatter (SSC) versus a FITC-conjugated CD14 mab. Cells were gated for live cells by forward scatter (FSC) and SSC. Purity of isolated CD14+ cells was generally ≥ 95%, in this example >99%. (B) Representative dot plot of immature moDCs with a PE-conjugated CD1a mab versus a FITC-conjugated CD209 (DC-SIGN) mab. Cells were gated for live cells by FSC and SSC. Approximately 90% of the cells were positive for CD1a and CD209. (C) Representative histograms of immature moDCs using the following mabs: APC/H7-conjugated HLA-DR antibody, PE-conjugated CD1a antibody, FITC-conjugated CD209 antibody, APC-conjugated CD83 antibody, PE/Cy7-conjugated CD80 antibody and PerCP/Cy5.5-conjugated CD86 antibody. Immature moDCs typically expressed high amounts of CD1a, CD209 as well as HLA-DR and low or intermediated amounts of CD80, CD83 as well as CD86. Red lines—unstained cells, green lines—stained cells. (TIF) [file ppat.1006643.s002.tif]

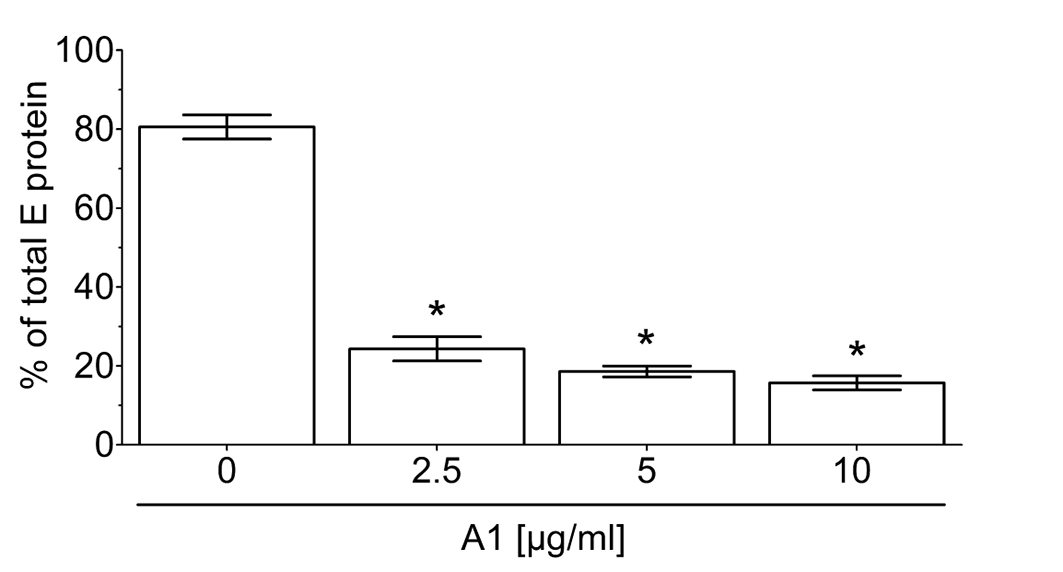

Supplement: S2 Fig — Mixtures of TBEV with mab A5 were incubated with increasing concentrations of the FL-specific mab A1 (0, 2.5, 5, 10 μg/ml) before incubation with liposomes for 1 hour at 37°C. The y-axis indicates percent bound virus relative to input virus using the amounts of E protein determined by quantitative ELISA. Data represent the mean +/- SEM of three independent experiments. The amounts of TBEV bound to liposomes in the presence of antibodies were compared to those obtained with the TBEV-A5 complex in the absence of these mabs (first column, 0 μg/ml) with ANOVA followed by Dunnett’s multiple comparisons test. *, p < 0.05. (TIF) [file ppat.1006643.s003.tif]

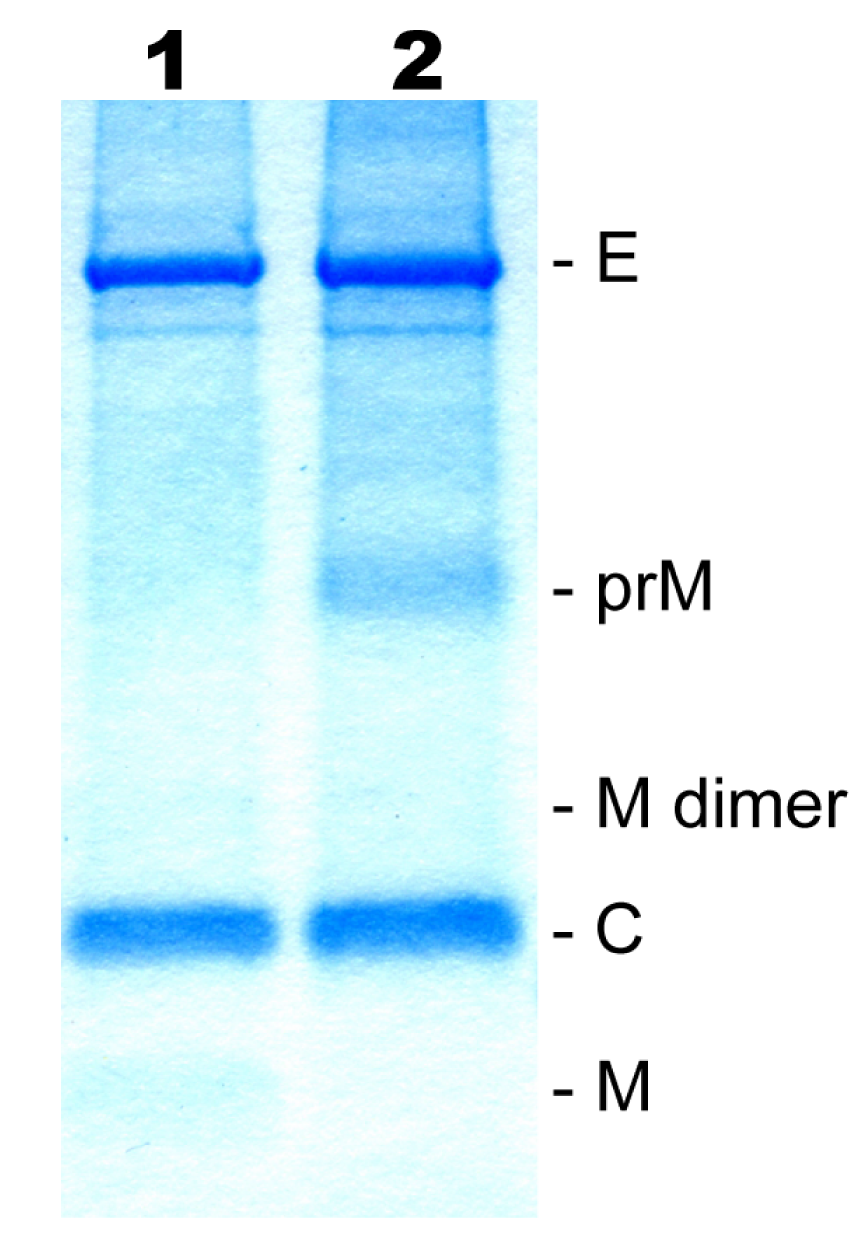

Supplement: S3 Fig — An aliquot of the purified TBEV preparation used in the present work (lane 1) was precipitated with trichloroacetic acid, subjected to SDS-PAGE and stained with Coomassie blue. A purified immature virus preparation obtained by growth in the presence of ammonium chloride was used as a control (lane 2). (TIF) [file ppat.1006643.s004.tif]

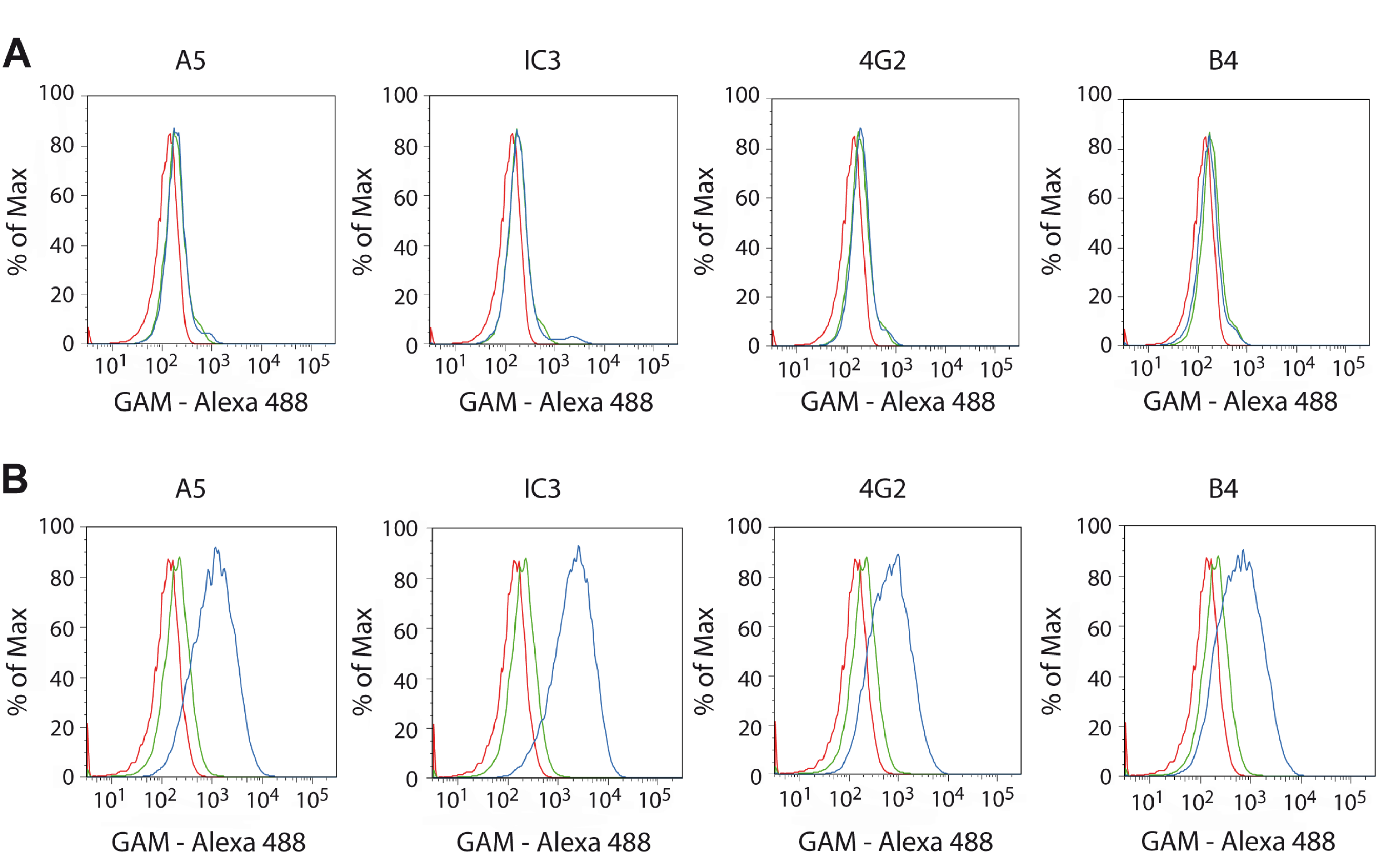

Supplement: S4 Fig — (A) Representative histograms of HeLa cells and mabs A5, IC3, 4G2 and B4. (B) Representative histograms of K562 cells and mabs A5, IC3, 4G2 and B4. Cells were gated for live cells by FSC and SSC. Red lines—unstained cells, green lines—cells stained with the Alexa Fluor 488-labeled anti-mouse conjugate, blue lines—cells stained with the mabs and the Alexa Fluor 488-labeled anti-mouse conjugate. (TIF) [file ppat.1006643.s005.tif]
